# Supplementary material for: Assessment of tuberculosis transmission probability in three Thai prisons based on five dynamic models
Source: PLoS One. 2024 Jul 19;19(7):e0305264. doi: 10.1371/journal.pone.0305264 (PMC11259261; doi:10.1371/journal.pone.0305264)
Supplement: S1 Fig — (DOCX) [file pone.0305264.s001.docx]

**S1 Figure**

Bland–Altman plots portraying the agreement pattern among the dynamic models

| **Difference** | **%Difference** |
| --- | --- |
| **Wells–Riley’s model versus Rudnick & Milton’s (ACH) model** | |
| (a)   | (b)   |
| **Wells–Riley’s model versus Rudnick & Milton’s (L/s/p) model** | |
| (c)   | (d)   |
| **Wells–Riley’s model versus Issarow *et al.*,’s model** | |
| (e)   | (f)   |
| **Wells–Riley’s model versus the applied SEIR model** | |
| (g)   | (h)   |
| **Rudnick & Milton’s (ACH) model versus Rudnick & Milton’s (L/s/p) model** | |
| (i)   | (j)   |
| **Rudnick & Milton’s (ACH) model versus Issarow *et al.*’s model** | |
| (k)   | (l)   |
| **Rudnick & Milton’s (ACH) model versus the applied SEIR model** | |
| (m)   | (n)   |
| **Rudnick & Milton’s (L/s/p) model versus Issarow *et al.*’s model** | |
| (o)   | (p)   |
| **Rudnick & Milton’s (L/s/p) model versus the applied SEIR model** | |
| (q)   | (r)   |
| **Issarow *et al.*’s model versus the applied SEIR model** | |
| (s)   | (t)   |
