# Supplementary material for: Assessment of tuberculosis transmission probability in three Thai prisons based on five dynamic models
Source: PLoS One. 2024 Jul 19;19(7):e0305264. doi: 10.1371/journal.pone.0305264 (PMC11259261; doi:10.1371/journal.pone.0305264)
Supplement: S1 Table — (DOCX) [file pone.0305264.s002.docx]

**S1 Table. Parameters used in prediction models**

1. **Cell architectural and environmental characteristics**

| **Parameters** | **Values**  **Med (IQR)** | **Units** | **References** |
| --- | --- | --- | --- |
| Floor area (length × width) | 16.44 (7.71–28.00) | m^2^ | Three Thai prisons, October 2020–May 2022 |
| Ceiling height | 3.50 (3.35–3.50) | m | Three Thai prisons, October 2020–May 2022 |
| Cell volume (length × width × height) | 55.08 (26.97–100.80) | m^3^ | Three Thai prisons, October 2020–May 2022 |
| The percentage of openings facing prevailing winds to the volume of a cell | 3.10 (2.83–3.49) | percent | Three Thai prisons, October 2020–May 2022 |
| The percentage of openings in the building to the volume of a cell | 4.02 (1.00–12.07) | percent | Three Thai prisons, October 2020–May 2022 |
| Number of ventilation fans | 1 (0–1) | unit | Three Thai prisons, October 2020–May 2022 |
| Ventilation fan area | 0.07 (0–0.13) | m^2^ | Three Thai prisons, October 2020–May 2022 |
| Number of cells per courtyard | 84 (48–191) | cells | Three Thai prisons, October 2020–May 2022 |
| Germ-free ventilation rate, Q in liters/second/person = 10^6^ × G/C _in, eq_– C_out_ [1, 2] | 25.00 (19.40–33.77) | L/s/p | Three Thai prisons, October 2020–May 2022 |
| G, the carbon dioxide generation rate in the space calculated from the average carbon dioxide generation rate per person at rest (0.0052 liters/second) and the number of occupants in the cell |  |  |  |
| C_in, eq_, indoor carbon dioxide concentration in parts per million (ppm) collected after the lock-up time of >13 hours |  |  |  |
| C _out_, outdoor carbon dioxide concentration in parts per million (ppm) collected after the lock-up time of >13 hours |  |  |  |
| Germ-free ventilation rate, *Q* in air changes per hour = [0.65 × wind speed (m/s) × opening facing prevailing winds (m^2^) × 3,600 s/h]/room volume (m^3^) [1] | 29.90 (18.82–45.96) | ACH |  |

1. **Demographics and health status of cell inmates**

| **Parameters** | **Values**  **Med (IQR)** | **Units** | **References** |
| --- | --- | --- | --- |
| Number of total and susceptible inmates per cell, S | 10 (5–24) | person | Three Thai prisons, October 2020–May 2022 |
| Room volume per person | 5.16 (4.38–5.47) | m^3^/person | Three Thai prisons, October 2020–May 2022 |
| Inmate turnover rate, Ω | 4.64 (1.39–9.66) | percent/year | Three Thai prisons, November 1, 2020–January 31, 2021 |
| Prisoners per building | 1,014 (982–1,118) | person | Three Thai prisons, October 2020–May 2022 |
| Residence time per day, Ө or t ^†^ | 15 | hour | Three Thai prisons, October 2020–May 2022 |

1. **Tuberculosis infection- and progression-related parameters**

| **Parameters** | **Values**  **[med (IQR)]** | **Units** | **References** |
| --- | --- | --- | --- |
| Prevalence of overall TB-infectious patients in the zone | 0.98 (0.49–1.72) | percent/180 days | The gathering of the secondary data was initiated in October 2020. Data was recorded as a percentage for Prisons A and B for a timeframe of 6 months, from July 2020 to December 2020. Meanwhile, data was recorded from October 2020 to March 2021 for Prison C. |
| Prevalence of smear-positive infectious patients in the zone | 0.51 (0.20–0.81) |  |  |
| Prevalence of smear-negative infectious patients in zone | 0.42 (0.30–0.91) |  |  |
| Number of TB-infectious patients in the cell | 0 (0–0) | persons/180 days |  |
| Number of smear-positive patients in the cell | 0 (0–0) |  |  |
| Number of smear-negative patients in the cell | 0 (0–0) |  |  |
| Infectious quanta rate, q ^†^  Infectious quanta rate, smear-positive ^†^  Infectious quanta rate, smear-negative ^†^ | 1  1.25    0.20 | quanta/hour | [3], [4], [5], [6],[7]  [8], [1]  [9], [1] |
| Transmission rate/ effective contact rate, β = PQ/VA | 0.029 (0. 016–0.048) | /persons/year | Wells–Riley equation [10–14] |
| Pulmonary ventilation rate of the susceptible, P ^†^ | 0.36 | m^3^/hour | [15] |
| Quanta production rate per infector, Q (rang) ^†^ | 12.7 (1.25–12.7) | quanta/hour or doses hr^−1^ | [3, 6] |
| Room volume per inmate, V | 5.16 (4.38–5.47) | m^3^ | Three Thai prisons, October 2020–May 2022 |
| Ventilation rate in air changes per hour, A | 29.90 (18.82–45.96) | ACH |  |
| Proportion of fast progressors (range) ^†^ | 0.14 (0.08–0.25) | percent | [16, 17] |
| Rate of fast progressors developing infectious TB (primary progression), σ_1_ (range) ^†^ | 0.9638 (0.76–0.99) | /year | [17, 18] |
| Rate of fast progressors moving to slow progressors, σ_2_ ^†^ | 0.2 | /year | [17, 19, 20] |
| Short-term latently infected, L1 or  SβI | 0.27 (0.10–1.33) | persons/year | Three Thai prisons, October 2020–May 2022 |
| S, the number of susceptible inmates |  |  |  |
| β, transmission rate |  |  |  |
| I, the number of infectious patients in each cell and zone |  |  |  |
| Rate of slow progressors developing infectious TB, ω (range) ^†^ | 0.00256 (0.00256–0.00527) | /year | [21] |
| Long-term latently infected ((1-p) L1 σ_2_), L2 | 0.05 (0.02–0.23) | persons/year | Three Thai prisons, October 2020–May 2022 |
| Relapse rate, r (range) ^†^ | 0.01 (0–0.03) | /year | [21] |
| Partial acquired immunity after primary infection for treated persons, ƒ (range) ^†^ | 0.41 (0.4–0.9) | percent | [17, 18] |
| Rate of recovery under anti-tuberculosis treatment, α ^†^ | 2 (as per guidelines, 6 months) | percent | [20, 22] |
| Natural recovery rate, α_n_ (range) ^†^ | 0.058 (0.021–0.086) | /year | [21, 23] |
| Surviving airborne infectious doses, β - µ ^†^ | 1–30 | doses hr ^−1^ | [24] |

1. **Physiological parameters**

| **Parameters** | **Values**  **Med (IQR)** | **Units** | **References** |
| --- | --- | --- | --- |
| Pulmonary ventilation rate, p ^†^ | 360 0.36 6 | liters/hour  m^3^/hour liters/minute | [15, 25] |
| A respiratory deposition fraction of airborne infectious particles that successfully reach and deposit at the target infectious site of the host for Issarow *et al*.’s model, Ө ^†^ | 0.1 | % | [26] |

1. **Treatment effectiveness parameters**

| **Parameters** | **Values**  **Med (IQR)** | **Units** | **References** |
| --- | --- | --- | --- |
| Infectiousness period (T or Ө for Rudnick & Milton-proposed model) | 116.39 (105.31–206.42) | day | Three Thai prisons, October 2020–May 2022 |
| Number of recovered patients, R ^†^ | 0.25 | percent/year | Three Thai prisons, October 2020–Sep 2021  (The proportion of treated cases who are cured was obtained form the follow up record of TB cases) |
| Untreated TB mortality rate, µ_1_ (range) ^†^ | 0.14 (0.058–0.461) | per year | [14, 21] |
| Natural/other mortality rates, µ^†^ | 0.15 | percent/year | Natural/other mortality rates of PTB case in three Thai prisons between October 2020–Sep 2021. |

† A constant variable was used in this study.

**References**

1. Urrego J, Ko AI, da Silva Santos Carbone A, Paião DS, Sgarbi RV, Yeckel CW, et al. The impact of ventilation and early diagnosis on tuberculosis transmission in Brazilian prisons. Am J Trop Med Hyg. 2015;93: 739-746. doi: 10.4269/ajtmh.15-0166.

2. Persily A. Evaluating building IAQ and ventilation with indoor carbon dioxide. ASHRAE Trans 103: 1–12. American Society of Heating, Refrigerating and Air-Conditioning Engineers (ASHRAE) annual meeting; 28 Jun–2 Jul 1997; Boston, MA (United States). United States1997. pp. 1-12.

3. Riley RL, Wells WF, Mills CC, Nyka W, McLean RL. Air hygiene in tuberculosis: quantitative studies of infectivity and control in a pilot ward. Am Rev Tuberc. 1957;75: 420-431. doi: 10.1164/artpd.1957.75.3.420.

4. Catanzaro A. Nosocomial tuberculosis. Am Rev Respir Dis. 1982;125: 559-562. doi: 10.1164/arrd.1982.125.5.559.

5. Noakes CJ, Sleigh PA. Mathematical models for assessing the role of airflow on the risk of airborne infection in hospital wards. J R Soc Interface. 2009;6: S791-S800. doi: 10.1098/rsif.2009.0305.focus.

6. Nardell EA, Keegan J, Cheney SA, Etkind SC. Airborne infection. Theoretical limits of protection achievable by building ventilation. Am Rev Respir Dis. 1991;144: 302-306. doi: 10.1164/ajrccm/144.2.302

7. Johnstone-Robertson S, Lawn SD, Welte A, Bekker LG, Wood R. Tuberculosis in a South African prison - a transmission modelling analysis. S Afr Med J. 2011;101: 809-813.

8. Riley RL, Mills CC, O'Grady F, Sultan LU, Wittstadt F, Shivpuri DN. Infectiousness of air from a tuberculosis ward. Ultraviolet irradiation of infected air: comparative infectiousness of different patients. Am Rev Respir Dis. 1962;85: 511-525. doi: 10.1164/arrd.1962.85.4.511.

9. Behr MA, Warren SA, Salamon H, Hopewell PC, Ponce de Leon A, Daley CL, et al. Transmission of Mycobacterium tuberculosis from patients smear-negative for acid-fast bacilli. Lancet. 1999;353: 444-449. doi: 10.1016/s0140-6736(98)03406-0.

10. Noakes CJ, Beggs CB, Sleigh PA, Kerr KG. Modelling the transmission of airborne infections in enclosed spaces. Epidemiol Infect. 2006;134: 1082-1091. doi: 10.1017/S0950268806005875.

11. Beggs CB, Noakes CJ, Sleigh PA, Fletcher LA, Siddiqi K. The transmission of tuberculosis in confined spaces: an analytical review of alternative epidemiological models. Int J Tuberc Lung Dis. 2003;7: 1015-526.

12. Gammaitoni L, Nucci MC. Using a mathematical model to evaluate the efficacy of TB control measures. Emerg Infect Dis. 1997;3: 335-342. doi: 10.3201/eid0303.970310.

13. Riley RL. Airborne infection. Am J Med. 1974;57: 466-475.

14. Tiemersma EW, van der Werf MJ, Borgdorff MW, Williams BG, Nagelkerke NJ. Natural history of tuberculosis: duration and fatality of untreated pulmonary tuberculosis in HIV negative patients: a systematic review. PLOS ONE. 2011;6: e17601. doi: 10.1371/journal.pone.0017601.

15. Guo Y, Qian H, Sun Z, Cao J, Liu F, Luo X, et al. Assessing and controlling infection risk with Wells-Riley model and spatial flow impact factor (SFIF). Sustain Cities Soc. 2021;67: 102719. doi: 10.1016/j.scs.2021.102719.

16. Dye C, Garnett GP, Sleeman K, Williams BG. Prospects for worldwide tuberculosis control under the WHO DOTS strategy. Directly observed short-course therapy. Lancet. 1998;352: 1886-1891. doi: 10.1016/s0140-6736(98)03199-7.

17. Vynnycky E, Fine PE. The natural history of tuberculosis: the implications of age-dependent risks of disease and the role of reinfection. Epidemiol Infect. 1997;119: 183-201. doi:10.1017/s0950268897007917.

18. Legrand J, Sanchez A, Le Pont F, Camacho L, Larouze B. Modeling the impact of tuberculosis control strategies in highly endemic overcrowded prisons. PLOS ONE. 2008;3: e2100. doi: 10.1371/journal.pone.0002100.

19. Dowdy DW, Dye C, Cohen T. Data needs for evidence-based decisions: a tuberculosis modeler's 'wish list'. Int J Tuberc Lung Dis. 2013;17: 866-877. doi: 10.5588/ijtld.12.0573.

20. Naning H, Al-Darraji HAA, McDonald S, Ismail NA, Kamarulzaman A. Modelling the impact of different tuberculosis control interventions on the prevalence of tuberculosis in an overcrowded prison. Asia Pac J Public Health. 2018;30: 235-243. doi: 10.1177/1010539518757229.

21. Blower SM, McLean AR, Porco TC, Small PM, Hopewell PC, Sanchez MA, et al. The intrinsic transmission dynamics of tuberculosis epidemics. Nat Med. 1995;1: 815-821. doi: 10.1038/nm0895-815.

22. Ministry of Health Malaysia, Academy of Medicine Malaysia. Clinical practice guidelines. Management of tuberculosis. 4, editor: Malaysian Health Technology Assessment Section (MaHTAS). Medical Development Division, Ministry of Health Malaysia; 2021. pp. 97.

23. Grzybowski S, Enarson DA. The fate of cases of pulmonary tuberculosis under various treatment programmes. Bull IUAT. 1978;53: 70-75.

24. Issarow CM, Mulder N, Wood R. Environmental and social factors impacting on epidemic and endemic tuberculosis: a modelling analysis. R Soc Open Sci. 2018;5: 170726. doi: 10.1098/rsos.170726.

25. Pinna GD, Maestri R, La Rovere MT, Gobbi E, Fanfulla F. Effect of paced breathing on ventilatory and cardiovascular variability parameters during short-term investigations of autonomic function. Am J Physiol Heart Circ Physiol. 2006;290: H424-H433. doi: 10.1152/ajpheart.00438.2005.

26. Issarow CM, Wood R, Mulder N. Seminal mycobacterium tuberculosis in vivo transmission studies: reanalysis using probabilistic modelling. Mycobact Dis. 2016;6: 217. doi: 10.4172/2161-1068.1000217.
