# Supplementary material for: Assessment of tuberculosis transmission probability in three Thai prisons based on five dynamic models
Source: PLoS One. 2024 Jul 19;19(7):e0305264. doi: 10.1371/journal.pone.0305264 (PMC11259261; doi:10.1371/journal.pone.0305264)
Supplement: S2 Table — (DOCX) [file pone.0305264.s003.docx]

**S2 Table. Models used in predicting the probability of tuberculosis transmission**

| **Dynamic models**  **Detail** | **The Wells–Riley equation [1, 2]** | **The Rudnick & Milton-proposed models [3, 4]** | **The Applied SEIR tuberculosis transmission model [5, 6]** | **The Issarow et al.**’s **models [7, 8]** |
| --- | --- | --- | --- | --- |
| Equation | C = S (1 − *e* *^− Ipqt/Q^*) | P = 1−exp [−Iqpt/Qө{1−V/Qө [1−exp (−Qө/V)]}] | dI/dt = Ω + pσ_1_*L_1_* + ω*L_2_* + rR – (α + α_n_)*I* - µ*I* - µ_1_*I* | P = 1–e^−^*^Pv(β − µ)Өpƒt^* |
| Equation variables | **C**, the number of TB infections  **S**, susceptible inmates  **I**, the number of TB-infectious patients  **p**, respiration rate (0.36 cubic meters per hour) [9]  **q**, quanta of infectious particles produced per hour (1 quanta/hour) [1]  **t**, time of exposure in minutes/lock-up time per day (hour)/infectiousness period [1, 10]  **Q**, germ-free ventilation (air changes per hour) [1] | **P**, the probability of infection in a confined space  **I**, the number of TB-infectious patients  **p**, respiratory rate (360 liters per hour or 0.36 cubic meters per hour) [9]  **Q**, infectious quanta rate, smear-positive (1.25 quanta/hour) [11] and infectious quanta rate, smear-negative (0.20 quanta/hour) [12]  **t**, infectiousness period (180 days) [13]  **V**, cell volume  **Ө**, occupied time cell (hours)  **Q**, germ-free ventilation rate (liters/second/person or air changes per hour) [3] | **S**, susceptible inmates  **L_1_**, short-term, latently infected  **L_2_**, long-term, latently infected  **I**, the number of TB-infectious patients  **R**, the number of recovered patients  **β**, TB transmission rate/the effective contact rate [2, 6, 14–16]  **p**, the proportion of fast progressors [17, 18]  **σ_1_**, rate of fast progressors developing infectious TB (primary progression) [17, 19]  **σ_2_**, rate of fast progressors moving to slow progressors [5, 17, 20]  **ω**, rate of slow progressors developing infectious TB [21]  **α_n_**, natural recovery rate [21, 22]  **α**, rate of recovery under the antituberculosis treatment [23] **µ,** Natural/other mortality rates **µ_1_**,TB mortality rate [16, 21]  **r**, relapse rate [21]  **ƒ**, partial acquired immunity after the primary infection for treated persons [17, 19, 24] **Ω**, inmate turnover rate  **τ_1_, τ_2_**, rate of recovery under preventive therapy for fast or slow progressors [23, 25] | **P**, the risk probability of TB transmission  **Pv**, the prevalence of infectors in the space [7]  **β-µ**, surviving airborne infectious dose per unit time (1–30 doses hr^−1^), where β is the total number of airborne infectious dose production per unit time and µ is the mortality rate of generated airborne infectious particles by the infector that do not reach the alveoli [8] **Ө**, alveoli deposition fraction [26]  **p**, respiration rate (0.36 cubic meters per hour) [9]  ***^ƒ^*or pn/Q**, p is the average pulmonary ventilation rate (0.36 cubic meters per hour), n is the susceptible inmate, and Q is the ventilation rate (ACH)  **t**, infectiousness period [7] |
|  |  |  |  |  |

**Reference**

1. Johnstone-Robertson S, Lawn SD, Welte A, Bekker LG, Wood R. Tuberculosis in a South African prison - a transmission modelling analysis. S Afr Med J. 2011;101(11):809-813.

2. Riley RL. Airborne infection. Am J Med 1974;57(3):466-475.

3. Urrego J, Ko AI, da Silva Santos Carbone A, Paião DS, Sgarbi RV, Yeckel CW, et al. The impact of ventilation and early diagnosis on tuberculosis transmission in Brazilian prisons. Am J Trop Med Hyg. 2015;93:739-746.

4. Rudnick SN, Milton DK. Risk of indoor airborne infection transmission estimated from carbon dioxide concentration. Indoor Air. 2003;13(3):237-245. doi: 10.1034/j.1600-0668.2003.00189.x.

5. Naning H, Al-Darraji HAA, McDonald S, Ismail NA, Kamarulzaman A. Modelling the impact of different tuberculosis control interventions on the prevalence of tuberculosis in an overcrowded prison. Asia Pac J Public Health. 2018;30(3):235-243. doi: 10.1177/1010539518757229.

6. Noakes CJ, Beggs CB, Sleigh PA, Kerr KG. Modelling the transmission of airborne infections in enclosed spaces. Epidemiol Infect. 2006;134(5):1082-1091. doi: 10.1017/S0950268806005875.

7. Issarow CM, Mulder N, Wood R. Modelling the risk of airborne infectious disease using exhaled air. J Theor Biol. 2015;372:100-106. doi: 10.1016/j.jtbi.2015.02.010.

8. Issarow CM, Mulder N, Wood R. Environmental and social factors impacting on epidemic and endemic tuberculosis: a modelling analysis. R Soc Open Sci. 2018;5(1):170726. doi: 10.1098/rsos.170726.

9. Pinna GD, Maestri R, La Rovere MT, Gobbi E, Fanfulla F. Effect of paced breathing on ventilatory and cardiovascular variability parameters during short-term investigations of autonomic function. Am J Physiol Heart Circ Physiol. 2006;290(1):H424-H433. doi: 10.1152/ajpheart.00438.2005.

10. Storla DG, Yimer S, Bjune GA. A systematic review of delay in the diagnosis and treatment of tuberculosis. BMC Public Health. 2008;8:15. doi: 10.1186/1471-2458-8-15.

11. Riley RL, Mills CC, O'Grady F, Sultan LU, Wittstadt F, Shivpuri DN. Infectiousness of air from a tuberculosis ward. Ultraviolet irradiation of infected air: comparative infectiousness of different patients. Am Rev Respir Dis. 1962;85:511-525. doi: 10.1164/arrd.1962.85.4.511.

12. Behr MA, Warren SA, Salamon H, Hopewell PC, Ponce de Leon A, Daley CL, et al. Transmission of Mycobacterium tuberculosis from patients smear-negative for acid-fast bacilli. Lancet. 1999;353(9151):444-449. doi: 10.1016/s0140-6736(98)03406-0.

13. Carbone S, Paião DS, Sgarbi RV, Lemos EF, Cazanti RF, Ota MM, et al. Active and latent tuberculosis in Brazilian correctional facilities: a cross-sectional study. BMC Infect Dis. 2015;15:24. doi: 10.1186/s12879-015-0764-8.

14. Beggs CB, Noakes CJ, Sleigh PA, Fletcher LA, Siddiqi K. The transmission of tuberculosis in confined spaces: an analytical review of alternative epidemiological models. Int J Tuberc Lung Dis 2003;7:1015-26.

15. Gammaitoni L, Nucci MC. Using a mathematical model to evaluate the efficacy of TB control measures. Emerg Infect Dis 1997;3:335-42.

16. Tiemersma EW, van der Werf MJ, Borgdorff MW, Williams BG, Nagelkerke NJ. Natural history of tuberculosis: duration and fatality of untreated pulmonary tuberculosis in HIV negative patients: a systematic review. PLOS ONE. 2011;6(4):e17601. doi: 10.1371/journal.pone.0017601.

17. Vynnycky E, Fine PE. The natural history of tuberculosis: the implications of age-dependent risks of disease and the role of reinfection. Epidemiol Infect. 1997;119(2):183-201. doi:10.1017/s0950268897007917.

18. World Health Organization. WHO consolidated guidelines on tuberculosis: Module 1: Prevention tuberculosis preventive treatment. Geneva: World Health Organization; 2020. 140 p.

19. Legrand J, Sanchez A, Le Pont F, Camacho L, Larouze B. Modeling the impact of tuberculosis control strategies in highly endemic overcrowded prisons. PLOS ONE. 2008;3(5):e2100. doi: 10.1371/journal.pone.0002100.

20. Dowdy DW, Dye C, Cohen T. Data needs for evidence-based decisions: a tuberculosis modeler's 'wish list'. Int J Tuberc Lung Dis. 2013;17(7):866-877. doi: 10.5588/ijtld.12.0573.

21. Blower SM, McLean AR, Porco TC, Small PM, Hopewell PC, Sanchez MA, et al. The intrinsic transmission dynamics of tuberculosis epidemics. Nat Med. 1995;1(8):815-821. doi: 10.1038/nm0895-815

22. Grzybowski S, Enarson DA. The fate of cases of pulmonary tuberculosis under various treatment programmes. Bull IUAT. 1978;53:70-75.

23. Ministry of Health Malaysia, Academy of Medicine Malaysia. Clinical practice guidelines. Management of tuberculosis. 4, editor: Malaysian Health Technology Assessment Section (MaHTAS). Medical Development Division, Ministry of Health Malaysia; 2021. 97 p.

24. Sutherland I, Svandová E, Radhakrishna S. The development of clinical tuberculosis following infection with tubercle bacilli. 1. A theoretical model for the development of clinical tuberculosis following infection, linking from data on the risk of tuberculous infection and the incidence of clinical tuberculosis in the Netherlands. Tubercle 1982;63(4):255-68.

25. World Health Organization. Guidelines for intensified tuberculosis case-finding and isoniazid preventive therapy for people liveing with HIV in resource-constrained settings. World Health 2011;01.

26. Issarow CM, Wood R, Mulder N. Seminal mycobacterium tuberculosis in vivo transmission studies: reanalysis using probabilistic modelling. Mycobact Dis. 2016;6(3):217. doi: 10.4172/2161-1068.1000217.
