# Supplementary material for: Assessment of tuberculosis transmission probability in three Thai prisons based on five dynamic models
Source: PLoS One. 2024 Jul 19;19(7):e0305264. doi: 10.1371/journal.pone.0305264 (PMC11259261; doi:10.1371/journal.pone.0305264)
Supplement: S3 Table — (DOCX) [file pone.0305264.s004.docx]

**S3 Table. Multiple linear regression analysis of the association between model parameters and changes in tuberculosis transmission probability predicted using five prediction models (n = 985)**

| **Cell characteristics** | **The Wells–Riley model** | | **The Rudnick & Milton-proposed model (ACH)** | | **The Rudnick & Milton-proposed model (L/s/p)** | | **The Applied SEIR TB transmission model** | | **Issarow *et al*.’s model** | |
| --- | --- | --- | --- | --- | --- | --- | --- | --- | --- | --- |
|  | **Δtp** | **(95% CI)** | **Δtp** | **(95% CI)** | **Δtp** | **(95% CI)** | **Δtp** | **(95% CI)** | **Δtp** | **(95% CI)** |
| **Architectural characteristics** |  | | | | | | | | | |
| **Cell volume (m^2^)** |  |  |  |  |  |  |  |  |  |  |
| **<30.00** |  |  | Ref. |  | Ref. |  |  |  |  |  |
| **30.01–60.00** |  |  | −0.02 | (−0.02, −0.01) | −0.03 | (−0.03, −0.02) |  |  |  |  |
| **60.01–120.00** |  |  | −0.05 | (−0.06, −0.04) | −0.05 | (−0.05, −0.04) |  |  |  |  |
| **> 120.00** |  |  | −0.08 | (−0.09, −0.07) | −0.03 | (−0.03, −0.02) |  |  |  |  |
| **Ventilation rate (ACH)** |  |  |  |  |  |  |  |  |  |  |
| **<12** | Ref. |  | Ref. |  |  |  | Ref. |  | Ref. |  |
| **12.01–30.00** | −0.14 | (−0.15, −0.12) | −0.05 | (−0.06, −0.04) |  |  | −0.04 | (−0.06, −0.02) | −0.11 | (−0.12, −0.09) |
| **30.01–45.00** | −0.18 | (−0.19, −0.16) | −0.08 | (−0.09, −0.06) |  |  | −0.07 | (−0.09, −0.04) | −0.13 | (−0.14, −0.11) |
| **45.01–60.00** | −0.20 | (−0.21, −0.18) | −0.09 | (−0.10, −0.08) |  |  | −0.11 | (−0.13, −0.08) | −0.14 | (−0.15, −0.12) |
| **> 60.00** | −0.20 | (−0.21, −0.18) | −0.10 | (−0.11, −0.09) |  |  | −0.07 | (−0.10, −0.04) | −0.15 | (−0.16, −0.13) |
| **Absolute ventilation rate (L/s/p) †** |  |  |  |  |  |  |  |  |  |  |
| **<15** |  |  |  |  | Ref. |  |  |  |  |  |
| **16–30** |  |  |  |  | −0.05 | (−0.06, −0.05) |  |  |  |  |
| **31–60** |  |  |  |  | −0.06 | (−0.07, −0.06) |  |  |  |  |
| **> 60** |  |  |  |  | −0.09 | (−0.10, −0.08) |  |  |  |  |
| **Demographic characteristics of cell inmates** |  | | | | | | | | | |
| **Number of inmates in the cell†** |  |  |  |  |  |  |  |  |  |  |
| **<5** | Ref.* |  | Ref.* |  | Ref.* |  | Ref. |  | Ref. |  |
| **6–10** | −0.03 | (−0.04, −0.02) | −0.03 | (−0.03, −0.02) | −0.02 | (−0.03, −0.02) | 0.04 | (0.02, 0.06) | −0.04 | (−0.05, −0.03) |
| **11–15** | −0.01 | (−0.03, 0.00) | −0.02 | (−0.03, −0.01) | −0.02 | (−0.03, −0.02) | 0.11 | (0.09, 0.13) | −0.03 | (−0.04, −0.02) |
| **16–20** | −0.04 | (−0.07, −0.01) | −0.04 | (−0.06, −0.03) | −0.04 | (−0.05, −0.02) | 0.14 | (0.09, 0.18) | −0.03 | (−0.06, 0.00) |
| **21–25** | 0.02 | (−0.04, 0.00) | −0.05 | (−0.06, −0.04) | −0.03 | (−0.04, −0.02) | 0.06 | (0.04, 0.07) | 0.01 | (−0.01, 0.02) |
| **> 25** | 0.00 | (−0.01, 0.02) | −0.06 | (−0.07, −0.05) | −0.03 | (−0.04, −0.03) | 0.12 | (0.10, 0.14) | 0.06 | (0.05, 0.08) |
| **Area per person (m^3^/person)** |  |  |  |  |  |  |  |  |  |  |
| **<3** | Ref.* |  | Ref.* |  | Ref.* |  | Ref. |  | Ref.* |  |
| **3.01–4.00** | −0.01 | (−0.04, 0.02) | 0.01 | (−0.01, 0.03) | 0.01 | (0.00, 0.03) | −0.11 | (−0.15, −0.08) | −0.05 | (−0.08, −0.03) |
| **4.01–5.00** | −0.03 | (−0.04, −0.01) | 0.04 | (0.03, 0.05) | 0.04 | (0.03, 0.05) | −0.17 | (−0.20, −0.15) | −0.06 | (−0.08, −0.04) |
| **5.01–6.00** | −0.01 | (−0.02, 0.01) | 0.06 | (0.04, 0.07) | 0.04 | (0.03, 0.06) | −0.20 | (−0.22, −0.18) | −0.05 | (−0.07, −0.03) |
| **> 6.00** | −0.03 | (−0.04, −0.01) | 0.05 | (0.03, 0.06) | 0.03 | (0.02, 0.05) | −0.19 | (−0.22, −0.17) | −0.07 | (−0.09, −0.05) |
| **Inmate turnover rate (%/year)** |  |  |  |  |  |  |  |  |  |  |
| **<2** |  |  |  |  |  |  | Ref. |  |  |  |
| **2.01–5.00** |  |  |  |  |  |  | 0.07 | (0.05, 0.08) |  |  |
| **5.01–10.00** |  |  |  |  |  |  | 0.11 | (0.09, 0.12) |  |  |
| **> 10** |  |  |  |  |  |  | 0.22 | (0.20, 0.23) |  |  |
| **Time-to-TB diagnosis in the cell (days)** |  |  |  |  |  |  |  |  |  |  |
| **<100** | Ref. |  | Ref. |  | Ref. |  | Ref.* |  | Ref. |  |
| **101–140** | −0.04 | (−0.05, −0.02) | −0.01 | (−0.03, 0.00) | −0.01 | (−0.02, 0.00) | 0.00 | (−0.02, 0.02) | −0.06 | (−0.07, −0.05) |
| **141–180** | 0.02 | (0.00, 0.03) | 0.01 | (0.00, 0.02) | 0.00 | (−0.01, 0.01) | 0.13 | (0.11, 0.15) | 0.00 | (−0.01, 0.01) |
| **> 180** | 0.08 | (0.07, 0.10) | 0.09 | (0.08, 0.10) | 0.05 | (0.04, 0.06) | 0.04 | (0.03, 0.06) | 0.02 | (0.01, 0.03) |
| **Number of overall TB cases in the cell** |  |  |  |  |  |  |  |  |  |  |
| **0** | Ref. |  | Ref. |  | Ref. |  | Ref. |  | Ref. |  |
| **1** | 0.03 | (0.02, 0.05) | 0.00 | (−0.01, 0.01) | 0.00 | (−0.01, 0.01) | 0.04 | (0.02, 0.06) | 0.03 | (0.02, 0.05) |
| **> 1** | 0.04 | (0.02, 0.06) | 0.02 | (0.00, 0.03) | 0.00 | (−0.01, 0.01) | 0.00 | (−0.03, 0.02) | 0.05 | (0.04, 0.07) |
| **Number of TB cases in the cell by smear status†** |  |  |  |  |  |  |  |  |  |  |
| **No** |  |  | Ref. |  | Ref. |  |  |  | Ref. |  |
| **Yes, but smear-negative** |  |  | 0.01 | (−0.01, 0.02) | 0.00 | (−0.01, 0.01) |  |  | 0.05 | (0.03, 0.06) |
| **Yes, and smear-positive** |  |  | 0.00 | (−0.01, 0.02) | 0.00 | (−0.01, 0.01) |  |  | 0.03 | (0.01, 0.05) |
| **Yes, and both smear-negative and smear-positive** |  |  | 0.02 | (0.01, 0.03) | 0.01 | (0.00, 0.02) |  |  | 0.05 | (0.03, 0.06) |
| **Prevalence of TB cases in the zone** |  |  |  |  |  |  |  |  |  |  |
| **Smear-negative TB cases** |  |  |  |  |  |  |  |  |  |  |
| **<0.30** |  |  | Ref. |  | Ref. |  |  |  | Ref. |  |
| **0.30–0.50** |  |  | −0.10 | (−0.11, −0.09) | −0.05 | (−0.05, −0.04) |  |  | −0.07 | (−0.09, −0.06) |
| **0.51–1.00** |  |  | 0.02 | (0.02, 0.03) | 0.02 | (0.02, 0.03) |  |  | 0.01 | (−0.01, 0.02) |
| **> 1.00** |  |  | 0.05 | (0.05, 0.06) | 0.02 | (0.01, 0.02) |  |  | 0.03 | (0.02, 0.04) |
| **Smear-positive TB cases** |  |  |  |  |  |  |  |  |  |  |
| **<0.20** |  |  | Ref. |  | Ref. |  |  |  | Ref. |  |
| **0.20–0.50** |  |  | 0.02 | (0.01, 0.04) | −0.02 | (−0.03, −0.01) |  |  | 0.01 | (−0.01, 0.02) |
| **0.51–0.80** |  |  | 0.06 | (0.05, 0.07) | 0.02 | (0.02, 0.02) |  |  | 0.03 | (0.01, 0.04) |
| **> 0.80** |  |  | 0.14 | (0.13, 0.15) | 0.07 | (0.06, 0.07) |  |  | 0.11 | (0.09, 0.12) |
| **Smear-negative and smear-positive TB cases** |  |  |  |  |  |  |  |  |  |  |
| **<0.50** | Ref. |  | Ref. |  | Ref. |  | Ref. |  | Ref. |  |
| **0.50–1.00** | 0.02 | (0.00, 0.03) | 0.03 | (0.02, 0.04) | 0.01 | (0.00, 0.01) | 0.11 | (0.10, 0.13) | 0.00 | (−0.01, 0.01) |
| **1.01–1.70** | 0.04 | (0.03, 0.05) | 0.06 | (0.05, 0.07) | 0.02 | (0.02, 0.03) | 0.02 | (0.01, 0.03) | 0.04 | (0.03, 0.05) |
| **> 1.70** | 0.20 | (0.19, 0.21) | 0.15 | (0.14, 0.16) | 0.07 | (0.06, 0.07) | 0.23 | (0.22, 0.24) | 0.09 | (0.07, 0.10) |

Δtp, magnitude of the change in the predicted TB transmission probability

* The parameter was not adjusted in the multiple linear regression model.
