# Supplementary material for: Assessment of tuberculosis transmission probability in three Thai prisons based on five dynamic models
Source: PLoS One. 2024 Jul 19;19(7):e0305264. doi: 10.1371/journal.pone.0305264 (PMC11259261; doi:10.1371/journal.pone.0305264)
Supplement: Attachment — Submitted filename: PONE-D-24-02877_reviewer-Copy.pdf [file pone.0305264.s005.pdf]

Wondershare PDFelement

PLOS ONE

Assessment of tuberculosis transmission probability in three Thai prisons based on five dynamic models

--Manuscript Draft--

**Manuscript Number:**	PONE-D-24-02877
**Article Type:**	Research Article
**Full Title:**	Assessment of tuberculosis transmission probability in three Thai prisons based on five dynamic models
**Short Title:**	Assessment of tuberculosis transmission probability in three Thai prisons based on five dynamic models
**Corresponding Author:**	Wiroj Jiamjarasrangsi, M.D.<br>Chulalongkorn University Faculty of Medicine<br>Bangkok, THAILAND
**Keywords:**	the probability of tuberculosis transmission; five dynamic models; the impact of model parameters; prisons
**Abstract:**	This study aimed to assess the probability of tuberculosis (TB) transmission based on five dynamic models: the Wells–Riley equation, two Rudnick & Milton-proposed models based on air changes per hour (ACH) and liters per second per person (L/s/p), the Applied Susceptible-Exposed-Infected-Recovered (SEIR) TB transmission model, and models proposed by Issarow et al. The study also aimed to determine the impact of model parameters on such probabilities in three Thai prisons. A cross-sectional study was conducted using data from 985 cells in the prisons. The TB transmission probability for each cell was calculated using parameters related to cell architectural and environmental characteristics, inmate demographics and health status, TB infection and progression, and physiological and treatment effectiveness. Subsequently, a multiple linear regression analysis was conducted to investigate the influence of each model parameter on the estimated probability. The results revealed that the median (Quartiles 1 and 3) of TB transmission probability among these cells was 0.052 (0.017, 0.180). The ventilation rate and the number of infectious TB patients in each cell or zone had the greatest impact on the estimated TB transmission probability in most models. Additionally, the number of inmates in each cell, the area per person in square meters, and the inmate turnover rate were identified as high-impact parameters in the Applied SEIR model. All stakeholders must urgently address these influential parameters to reduce TB transmission in prisons. In addition, further studies are required to determine their relative validity in accurately predicting TB incidence in prison settings.
**Order of Authors:**	Nithinan - Mahawan, Ph.D candidate
	Thanapoom Rattananupong
	Puchong Sri-Uam
	Wiroj Jiamjarasrangsi
**Additional Information:**	
**Question**	**Response**
**Financial Disclosure**<br><br>Enter a financial disclosure statement that describes the sources of funding for the work included in this submission. Review the submission guidelines for detailed requirements. View published research articles from *PLOS ONE* for specific examples.	- WJ and NM received the 90th Anniversary of Chulalongkorn University Fund (grant no. 035, 1/2021) and the FY2021 Thesis Grant for Doctoral Degree Study of the National<br>Research Council of Thailand (NRCT; grant no. N41D640002, 2021).<br>- URI (the 90th Anniversary of Chulalongkorn University Fund):<br>https://www.grad.chula.ac.th/index.php?lang=th<br>-URI: https://www.nrct.go.th/<br>-No, the funders had no role in study design, data collection and analysis, decision to publish, or preparation of the manuscript.

Powered by Editorial Manager® and ProduXion Manager® from Aries Systems Corporation

Wondershare
PDFelement

This statement is required for submission and **will appear in the published article** if the submission is accepted. Please make sure it is accurate.<br><br>**Unfunded studies**<br>Enter: *The author(s) received no specific funding for this work.*<br><br>**Funded studies**<br>Enter a statement with the following details:<br>• Initials of the authors who received each award<br>• Grant numbers awarded to each author<br>• The full name of each funder<br>• URL of each funder website<br>• Did the sponsors or funders play any role in the study design, data collection and analysis, decision to publish, or preparation of the manuscript?<br>• **NO** - Include this sentence at the end of your statement: *The funders had no role in study design, data collection and analysis, decision to publish, or preparation of the manuscript.*<br>• **YES** - Specify the role(s) played.<br><br>* typeset	
**Competing Interests**<br><br>Use the instructions below to enter a competing interest statement for this submission. On behalf of all authors, disclose any competing interests that could be perceived to bias this work—acknowledging all financial support and any other relevant financial or non-financial competing interests.<br><br>This statement is required for submission and **will appear in the published article** if the submission is accepted. Please make sure it is accurate and that any funding sources listed in your Funding Information later in the submission form are also declared in your Financial Disclosure statement.<br><br>View published research articles from	The authors have declared that no competing interest exist.

Wondershare
PDFelement

Wondershare PDFelement

PLOS ONE for specific examples.<br><br>**NO authors have competing interests**<br><br>Enter: *The authors have declared that no competing interests exist.*<br><br>**Authors with competing interests**<br><br>Enter competing interest details beginning with this statement:<br><br>*I have read the journal's policy and the authors of this manuscript have the following competing interests: [insert competing interests here]*<br><br>* typeset	
**Ethics Statement**<br><br>Enter an ethics statement for this submission. This statement is required if the study involved:<br><br>• Human participants<br>• Human specimens or tissue<br>• Vertebrate animals or cephalopods<br>• Vertebrate embryos or tissues<br>• Field research<br><br>Write "N/A" if the submission does not require an ethics statement.<br><br>General guidance is provided below. Consult the submission guidelines for detailed instructions. **Make sure that all information entered here is included in the Methods section of the manuscript.**	(Date of Approval September 24, 2020 and Approval Expire Date September 23, 2021 1) Assistant<br>Professor Apichai Vasuratna, MD. // Vice-Chairman, Acting Chaiman, the Institutional Review Board.<br>2) Assistant Professor Patou Tantbirojn MD// Member and Assistant Secretary, Acting Secretary, the<br>Institutional Review Board).<br>((Date of Approval September 24, 2021 (First Extension) and Approval Expire Date September 23,<br>2022 1) Emeritus Professor Tada Sueblinvong, MD. // Chairperson, the Institutional Review Board. 2)<br>Assistant Professor Thananya Thongtan, PhD// Member and Secretary, the Institutional Review<br>Board)).

Wondershare PDFelement

Wondershare PDFelement

**Format for specific study types**<br><br>**Human Subject Research (involving human participants and/or tissue)**<br>• Give the name of the institutional review board or ethics committee that approved the study<br>• Include the approval number and/or a statement indicating approval of this research<br>• Indicate the form of consent obtained (written/oral) or the reason that consent was not obtained (e.g. the data were analyzed anonymously)<br><br>**Animal Research (involving vertebrate animals, embryos or tissues)**<br>• Provide the name of the Institutional Animal Care and Use Committee (IACUC) or other relevant ethics board that reviewed the study protocol, and indicate whether they approved this research or granted a formal waiver of ethical approval<br>• Include an approval number if one was obtained<br>• If the study involved *non-human primates*, add *additional details* about animal welfare and steps taken to ameliorate suffering<br>• If anesthesia, euthanasia, or any kind of animal sacrifice is part of the study, include briefly which substances and/or methods were applied<br><br>**Field Research**<br><br>Include the following details if this study involves the collection of plant, animal, or other materials from a natural setting:<br>• Field permit number<br>• Name of the institution or relevant body that granted permission	
**Data Availability**<br><br>Authors are required to make all data underlying the findings described fully available, without restriction, and from the time of publication. PLOS allows rare exceptions to address legal and ethical concerns. See the PLOS Data Policy and FAQ for detailed information.	Yes - all data are fully available without restriction

Wondershare PDFelement

Wondershare PDFelement

A Data Availability Statement describing where the data can be found is required at submission. Your answers to this question constitute the Data Availability Statement and **will be published in the article**, if accepted.<br><br>**Important:** Stating 'data available on request from the author' is not sufficient. If your data are only available upon request, select 'No' for the first question and explain your exceptional situation in the text box.<br><br>Do the authors confirm that all data underlying the findings described in their manuscript are fully available without restriction?	
**Describe where the data may be found in full sentences. If you are copying our sample text, replace any instances of XXX with the appropriate details.**<br><br>• If the data are **held or will be held in a public repository**, include URLs, accession numbers or DOIs. If this information will only be available after acceptance, indicate this by ticking the box below. For example: *All XXX files are available from the XXX database (accession number(s) XXX, XXX.*).<br>• If the data are all contained **within the manuscript and/or Supporting Information files**, enter the following: *All relevant data are within the manuscript and its Supporting Information files.*<br>• If neither of these applies but you are able to provide **details of access elsewhere**, with or without limitations, please do so. For example:<br><br>*Data cannot be shared publicly because of [XXX]. Data are available from the XXX Institutional Data Access / Ethics Committee (contact via XXX) for researchers who meet the criteria for access to confidential data.*<br><br>*The data underlying the results presented in the study are available from (include the name of the third party*	Data cannot be shared publicly because of security reasons of prisons. Data are available from documents and computer databases of the corresponding zonal and central administrative and medical facilities in the prisons./ Ethics Committee (contact via 610/63) for researchers who meet the criteria for access to confidential data.

Wondershare PDFelement

Wondershare
PDFelement

and contact information or URL).<br>• This text is appropriate if the data are owned by a third party and authors do not have permission to share the data.<br><br>* typeset	
Additional data availability information:	Tick here if the URLs/accession numbers/DOIs will be available only after acceptance of the manuscript for publication so that we can ensure their inclusion before publication.

Wondershare
PDFelement

Manuscript

Click here to access/download;Manuscript;28_Jan_24_M

Wondershare PDFelement

Research Article

Assessment of tuberculosis transmission probability in three Thai prisons based on five dynamic models

Nithinan Mahawan[1], Thanapoom Rattananupong[1], Puchong Sri-Uam[2], Wiroj Jiamjarasrangsi[1*]

[1] Department of Preventive and Social Medicine, Faculty of Medicine, Chulalongkorn University, Bangkok, Thailand

[2] Center for Safety, Health and Environment of Chulalongkorn University, Bangkok, Thailand.

[*]Corresponding author

E-mail: wjiamja@gmail.com (WJ)

Wondershare PDFelement

Wondershare
PDFelement

Data Availability Statement

All relevant data are included within the manuscript and its Supporting Information files.

Funding

This study was supported by the 90th Anniversary of Chulalongkorn University Fund (grant no. 035, 1/2021) and the FY2021 Thesis Grant for Doctoral Degree Study of the National Research Council of Thailand (NRCT; grant no. N41D640002, 2021).

Competing interests

The authors declare that they have no competing interests.

Abstract

This study aimed to assess the probability of tuberculosis (TB) transmission based on five dynamic models: the Wells–Riley equation, two Rudnick & Milton-proposed models based on air changes per hour (ACH) and liters per second per person (L/s/p), the Applied Susceptible-Exposed-Infected-Recovered (SEIR) TB transmission model, and models proposed by Issarow *et al*. The study also aimed to determine the impact of model parameters on such probabilities in three Thai prisons. A cross-sectional study was conducted using data from 985 cells in the prisons. The TB transmission probability for each cell was calculated using parameters related to cell architectural and environmental characteristics, inmate demographics and health status, TB infection and progression, and physiological and treatment effectiveness. Subsequently, a multiple linear regression analysis was conducted to investigate the influence of each model parameter on the estimated probability. The results revealed that the median (Quartiles 1 and 3) of TB transmission probability among these cells was 0.052 (0.017, 0.180). The ventilation rate and the number of infectious TB patients in each cell or zone had the greatest impact on

Wondershare
PDFelement

Wondershare PDFelement

the estimated TB transmission probability in most models. Additionally, the number of inmates in each cell, the area per person in square meters, and the inmate turnover rate were identified as high-impact parameters in the Applied SEIR model. All stakeholders must urgently address these influential parameters to reduce TB transmission in prisons. In addition, further studies are required to determine their relative validity in accurately predicting TB incidence in prison settings.

Introduction

Tuberculosis (TB) is the leading cause of illness and death from communicable diseases worldwide [1]. This is also applicable to Thailand, which is one of 14 countries with the highest prevalence of TB, TB/human immunodeficiency virus (HIV), and multidrug-resistant tuberculosis (MDR-TB) [1]. Although global TB incidence declined by about 9% from 2015 to 2019, one challenge is the high TB incidence among high-risk populations, such as prisoners, where TB incidence is 5 to 70 times higher than in the general population [2, 3]. Since Thailand has the 7th highest number of prisoners (incarcerated individuals) in the world [4], TB incidence in prisons is of particular public health concern in its attempt to meet the World Health Organization's (WHO) End TB Strategy milestone [3].

Wondershare PDFelement

High TB risk among prisoners is caused by environmental factors, such as overcrowding, limited access to healthcare, insufficient access to nutrition, poor ventilation, indoor and unsanitary confinement, disruption of social and support networks, and frequent movement within the carceral system. Additionally, personal risk factors, such as HIV infection status, cigarette smoking, illegal drug use, and limited access to healthcare before incarceration, contribute to the increased risk of TB among prisoners [5]. However, the prison environment, rather than host risk factors, is the main driver of TB risk [5]. This is supported by an observational and modeling study in Brazil that found that the incidence of latent TB was low

Wondershare PDFelement

at the time of incarceration, increased during incarceration, and declined after release to that of the general population over a seven-year period [6]. To reduce TB incidence among incarcerated individuals, specific aspects of the prison environment should therefore be targeted. Since prisons often act as institutional amplifiers of TB, implementing ameliorating measures will result in less TB spillover into the general population [6].

Currently, a few dynamic models of TB transmission have been applied in prison settings based on prison environmental features. These models include the Wells–Riley model [7], Rudnick & Milton-proposed models [8, 9], the Applied Susceptible-Exposed-Infected-Recovered (SEIR) TB transmission model [10], and models proposed by Issarow *et al*. [11, 12]. Each of these models predicts the probability of TB transmission based on different prison environmental factors, although some factors are shared by all of them. All of these models have the potential to identify prison environmental features with high leverage as intervention targets to reduce TB transmission. However, their comparability and validity in predicting future TB incidence have yet to be investigated.

Wondershare PDFelement

This study aimed to assess the probability of TB transmission in three Thai prisons using the five previously mentioned dynamic models and to determine the impact of model parameters such as prison architectural and environmental features, TB management, and inmates' demographics and TB-related characteristics on TB transmission probability. The ability of these dynamic models to predict future TB incidence among incarcerated individuals, i.e., predictive validity, will be reported in a separate article.

Materials and methods

Study population

Wondershare PDFelement

Mention the type os sampling method

A cross-sectional study was conducted in three Thai prisons: one supermaximum prison (Prison A, with five zones, 239 cells, and 4,734 inmates), one maximum prison (Prison B, with six zones, 652 cells, and 6,607 inmates) in Bangkok, and one maximum prison (Prison C, with four zones, 94 cells, and 6,216 inmates) in Rayong province, approximately 180 kilometers east of Bangkok. In 2021, the reported average occupancy rates of these prisons were 136.44%, 120%, and 145.40%, respectively, implying that the average cell floor area is less than the WHO recommendation of 5.40 m^2 floor area per inmate [13]. Each cell serves as the study and analysis unit, and a total of 985 cells from these prisons were included in this study. The study was approved by the Ethical Review Board of Chulalongkorn University Faculty of Medicine (IRB reference no. 610/63), and permission was obtained from the Department of Corrections at the Ministry of Justice before conducting the study. Before commencing the study, the superintendent of each correctional facility perused the "Information Sheet for Participants" and signed the "Submission Agreement for Volunteers." The study did not involve the collection of personal information that could lead to the identification of inmates, thus consent from individual inmates was not sought.

Wondershare PDFelement

Data collection

The following variables were used to calculate the probability of TB transmission for each cell: (1) Cell architectural and environmental characteristics, such as cell ventilation, room volume, outdoor air exchange rate, opening facing prevailing winds, opening into the building, and number of cells per courtyard; (2) the demographic and health status composition of inmates, including the number of total and susceptible inmates per cell, residence time per day, and inmate turnover; (3) TB infection- and progression-related parameters, such as the number of infectious patients, infectious quanta rate, surviving airborne infectious doses, transmission

Wondershare PDFelement

rate, proportion of fast progressors, rate of fast progressors developing infectious TB (primary progression), rate of fast progressors moving to slow progressors, short-term latently infected, rate of slow progressors developing infectious TB (reactivation), long-term latently infected, relapse rate, partial acquired immunity after primary infection for treated persons, rate of recovery under antituberculosis treatment, and natural recovery; (4) physiological parameters, including pulmonary ventilation rate and respiratory deposition fraction of airborne infectious particles; and (5) treatment effectiveness parameters, including period of infectiousness, number of recovered patients, and TB-related, natural, and other mortality rates. These variables and parameters were collected or obtained as shown in S1 Table.

Cell architectural and environmental characteristics

For security reasons, five inmates from each zone were recruited and trained by the principal investigator (NM) and a coinvestigator (PS, who is also an industrial hygienist) to survey the architectural and environmental characteristics of each cell within the zone. The data collected included floor area (m^2) and ceiling height (m) to calculate cell volume (V; m^3), size of open windows and doors (m^2), number of cells per courtyard, presence of cross-ventilation design (yes or no), temperature (°C), relative humidity (%), and the number and size of portable fans. In addition, the trained inmate team measured the area of the courtyard (m^2) and the outdoor wind speed (meters/second).

Wondershare PDFelement

Ventilation rate

As in previous studies, two methods were used to measure the cell ventilation rate in the real world, including the absolute ventilation rate (liters/second/person) and air changes per hour (ACH) [7, 8, 10, 11]. In Method 1, the absolute ventilation rate, always used as a surrogate for exhaled air, was assessed by steady-state carbon dioxide (CO_2) in parts per million using the

Wondershare PDFelement

Kimo HQ210 with SCOH 112 probe (Sauermann Industries, ZA Bernard Moulinet, Montpon, France). The CO_2 concentrations in the morning after at least 13 hours of lockup time within and outside each cell were measured by the trained inmate team [8, 9, 11]. The absolute ventilation rate (Q; L/s) for each cell was then calculated based on the equilibrium between ventilation and CO_2 production through respiration, according to Persily's model [14]. In Method 2, ACH (Q; ACH), as a rule of thumb, is classically used as a metric for assessing infection control risk. It is the total air volume in a room or space that is completely removed and replaced in an hour [15]. In this method, the wind speed (meters/second) in each cell was measured at the location of the opening facing prevailing winds using a hot wire thermo-anemometer and datalogger (Model SDL350, Extech Instruments, Waltham, MA). The credibility of the data was cooperatively ensured by the principal investigator (NM) and a coinvestigator (PS). Wind speed data was then combined with data on cell volume and the size of openings facing prevailing winds to calculate the ACH according to the WHO-proposed model [15].

Wondershare PDFelement

Inmate demographics and health status

Data on the number of all and susceptible inmates with HIV or chronic disease and aged 60 or older (S; in persons), residence time per day (ϴ or t; in hours), and inmate turnover rate (Ω; in percent per year) were obtained from primary data sources, such as surveys or interviews with staff and inmates, and secondary data sources, such as reports or computer databases corresponding to each zone in the prisons.

Physiological parameters

Data on the adult pulmonary ventilation rate (p; in liters/hour or m^3/hour) [16, 17] and the deposition fraction of airborne infectious particles that successfully reach the target infectious

Wondershare PDFelement

site of the host respiratory system (ϴ; in percent per surviving airborne infectious doses) [18] were obtained from published studies.

TB infection- and progression-related parameters

The data were obtained from documents and computer databases of the corresponding zonal and central administrative and medical facilities in the prisons. These data included the number of TB-infectious patients in each cell and zone, representing the incidence at the cell and zonal levels. This data was recorded as a percentage per 6 months during July–December 2020 for Prisons A and B, and during October 2020–March 2021 for Prison C. The TB infection- and progression-related parameters were obtained from relevant international literature. These parameters included data on the quanta of infectious particles produced per hour (q; in quanta/hour) [7, 8, 19–24], surviving airborne infectious doses per hour (β-μ; in doses hr^{-1}) [12], the proportion of fast progressors (p; in percent) [25, 26], the rate of fast progressors developing infectious TB (σ_1; per year) [25, 27], the rate of fast progressors moving to slow progressors (σ_2; per year) [25, 28], the rate of slow progressors developing infectious TB (ω; per year) [29], the relapse rate (r; per year) [29], partial acquired immunity after primary infection for treated persons (*f*; percent) [25, 27], the rate of recovery under antituberculosis treatment (α; percent) [10, 30], and the natural recovery rate (α_n; per year) [29, 31]. However, some TB infection- and progression-related parameters relied on data from three Thai prisons. These parameters included the TB transmission rate (β; per person per year), calculated using the formula PQ/VA, where P is the average pulmonary ventilation rate (0.36 m^3/hour), Q is the quanta production rate per infector (12.7 quanta/hour), V is the room volume per inmate (m^3), and A is the ventilation rate (ACH) [32]; the numbers of short-term latently infected (L1; in persons per year), calculated using the formula SβI, where S is the number of susceptible inmates, β is the TB transmission rate, and I is the number of TB-infectious patients in each

Wondershare PDFelement

Wondershare PDFelement

cell and zone (prevalence); and long-term infected individuals (L2; in persons per year), calculated using the formula (1-p) L1 σ_2, where p is the proportion of fast progressors [25, 26], L1 is the number of short-term latently infected, and σ_2 is the rate of fast progressors moving to slow progressors [25, 28].

Treatment effectiveness parameters

According to previous studies, the average period of infectivity or time exposure prior to diagnosis is 180 days [33, 34]. In this study, however, the average infectivity period (t; in days) or "time-to-TB diagnosis" from the studied prisons' database (i.e., inferring from the time-lapse from the date of annual chest x-ray TB surveillance, which differed for each zone of the prison, to the date of TB treatment initiated for each diagnosed inmate) was calculated. The number of recovered patients (R; in percent per year) and natural/other mortality rates (μ; in percent per year) were also obtained from secondary data collected in three Thai prisons. In addition, data on the TB-related death rate (μ_1; in percent per year) [29, 35] was obtained from the published study.

Wondershare PDFelement

TB transmission probability modeling

To calculate the probability of TB transmission, five dynamic models were used: the Wells–Riley equation [7], two Rudnick & Milton-proposed models based on ACH and liters/second/person (L/s/p) as ventilation rate parameters, respectively [8, 9], the SEIR TB transmission model [10], and models proposed by Issarow *et al*. [11, 12] (see S2 Table for more detail).

Statistical analysis

Wondershare
PDFelement

Cells were divided into three to six categories based on architectural and environmental characteristics, primarily using quartiles with modifications for meaningful and mutually exclusive categories as appropriate to ensure sufficient samples in each category. For categorical variables, the data were summarized as frequencies and percentages, while medians and interquartile ranges (IQR) were used for continuous variables. The subgroups were compared using appropriate statistical tests. For categorical data, the chi-squared test or Fisher's exact test was used when more than 20% of cells had expected frequencies less than 5. However, nonparametric tests such as the Wilcoxon rank–sum (Mann–Whitney) test were used for continuous, non-normally distributed data.

To investigate the influence of model parameters on TB transmission probability, two procedures were used separately for each TB transmission prediction model. First, cells were categorized into four subgroups based on the quartile of predicted TB transmission probability. Furthermore, subgroups of model parameters were then compared using the Wilcoxon rank–sum (Mann–Whitney) test since the cell-specific probabilities of TB transmission were non-normally distributed. Second, the magnitude and pattern of change in the predicted TB transmission probability along each model's parameter categories were investigated using a multiple linear regression incorporating all model parameters simultaneously for a specific prediction model. The adjusted beta (i.e., the magnitude of change in the predicted TB transmission probability) and its 95% confidence intervals were estimated. All statistical analyses were performed using Stata software, Version 13.0 (StataCorp. 2013, Stata Statistical Software: Release 13, College Station, TX: StataCorp LP).

Wondershare
PDFelement

Results

Wondershare PDFelement

Cell characteristics

In terms of the architectural characteristics of the two prisons (Prisons A and B) in Bangkok (the capital city), cells in Prison A had a larger average floor area (28.00 *vs.* 7.71 m^2), a higher ceiling (3.60 *vs.* 3.48 m), and a much larger cell volume (100.80 *vs.* 26.98 m^3) than those in Prison B (Table 1). However, cells in Rayong province's Prison C had a much larger average floor area (41.36 m^2) but a lower ceiling height (2.80 m) than those in Bangkok's prisons, with a larger cell volume (115.81 m^3). In terms of ventilation (Table 1), cells in Prison C had the highest average ventilation rates (39.93 ACH and 33.99 L/s/p), while cells in Prison A and B had comparable ventilation rates (29.81 *vs.* 28.93 ACH and 24.41 *vs.* 24.53 L/s/p).

The average number of inmates in cells was highest in Prison C (55 persons), followed by Prison A (23 persons), and lowest in Prison B (six persons), with a corresponding reverse pattern of area per person (2.16 *vs.* 4.38 *vs.* 5.20 m^3/person). The cell inmate turnover rates were highest in Prison C (40.30% per year), much lower in Prison A (8.40 % per year), and lowest in Prison B (1.69% per year).

Wondershare PDFelement

During the past six months, there was at least one TB case in 39 out of 239 cells (16.32%) in Prison A, 55 out of 652 cells (8.44%) in Prison B, and 35 out of 94 cells (37.23%) in Prison C. However, when considering the presence of TB cases in the zone as a potential source of TB exposure for inmates in each cell, the prevalence of infectious patients in the zone during the past six months was highest in Prison B (1.32%), followed by Prison A (0.98%), and Prison C (0.90%) (Table 1). Furthermore, the average time-to-TB diagnosis, calculated as the time from the date of getting ill to the date of formal TB diagnosis, was the longest in Prison A (365.21 days), followed by Prison C (161.93 days) and Prison B (116.39 days).

Wondershare PDFelement

Table 1. Cell architectural, environmental, and tuberculosis risk characteristics in the three prisons studied.

Local parameter	Formula					Prison A (*n* = 239)		Prison B (*n* = 652)		*p*	Prison C (*n* = 94)		*p*
	M1	M2	M3	M4	M5	Med	(Q1, Q3)	Med	(Q1, Q3)		Med	(Q1, Q3)	
**Architectural characteristics**													
**Floor area (m^2)** †	-	-	-	-	-	28.00	(28.00, 28.00)	7.71	(7.71, 16.44)	a	41.36	(41.36, 41.36)	a, b
**Ceiling height (m)** †	-	-	-	-	-	3.60	(3.60, 3.60)	3.48	(3.35, 3.50)	a	2.80	(2.80, 2.80)	a, b
**Cell volume (m^3)** †	-	√	√	-	-	100.80	(100.80, 100.80)	26.98	(25.82, 57.22)	a	115.81	(115.81, 115.81)	a, b
**Percent of the opening area in walls**													
**To prevailing wind (%)** †	-	-	-	-	-	3.93	(3.93, 3.93)	2.99	(2.60, 3.11)	a	2.05	(2.05, 3.95)	a, b
**To building (%)** †	-	-	-	-	-	15.18	(15.18, 15.18)	1.05	(1.00, 4.02)	a	5.67	(0, 5.67)	a, b
**Number of ventilation fans**†	-	-	-	-	-	1	(1, 1)	1	(0, 1)	a	0	(0, 1)	a, b
**Ventilation rate (ACH)** †	√	√	-	√	√	29.81	(23.40, 39.25)	28.93	(17.24, 46.86)	-	39.93	(21.52, 64.59)	a, b
**Absolute ventilation rate (L/s/p)** †	-	-	√	-	-	24.41	(18.57, 35.14)	24.53	(19.19, 30.86)	-	33.99	(24.64, 48.6)	a, b
**Demographics and health status of cell inmates**													
**Number of inmates in the cell** †	√	√	√	√	√	23	(13, 25)	6	(5, 11)	a	55	(46, 60)	a, b
**Area per person (m^3/person)** †	-	-	-	√	-	4.38	(4.03, 5.60)	5.20	(4.77, 5.63)	a	2.16	(1.93, 2.52)	a, b

Wondershare PDFelement

Local parameter	Formula M1	M2	M3	M4	M5	Prison A (*n* = 239) Med	(Q1, Q3)	Prison B (*n* = 652) Med	(Q1, Q3)	*p*	Prison C (*n* = 94) Med	(Q1, Q3)	*p*
**Inmate turnover rate (%/year)** †	-	-	-	√	-	8.40	(5.69, 9.54)	1.69	(1.39, 6.86)	a	40.30	(35.11, 41.50)	a, b
**Time to diagnosis TB cases in the cell (days)**†	√	√	√	-	√	365.21	(298.56, 446.53)	116.39	(70.85, 116.39)	a	161.93	(151.81, 161.93)	a, b
**Number of overall TB cases in the cell (prevalence in 180 days) [n (%)]** ‡	√	-	-	√	-					a			a, b
**0**						200	(83.68)	597	(91.56)		59	(62.77)	
**1**						30	(12.55)	20	(3.07)		20	(21.28)	
**> 1**						9	(3.77)	35	(5.37)		15	(15.96)	
**The prevalence of infectious patients in the cell (prevalence per 180 days)** †	√	-	-	√	-	0	(0, 0)	0	(0, 0)	a	0	(0, 0.02)	a, b
**The prevalence of infectious patients in the zone (prevalence per 100 persons per 180 days)** †	√	-	-	√	-	0.98	(0.53, 1.72)	1.32	(0.49, 1.32)	-	0.90	(0.57, 1.12)	-
**Number of TB cases in the cell by smear status [n (%)]** ‡	-	√	√	-	√					a			a, b
**No**						200	(83.68)	597	(91.56)		59	(62.77)	

Wondershare PDFelement

Wondershare PDFelement

Local parameter	Formula					Prison A ($n = 239$)		Prison B ($n = 652$)		p	Prison C ($n = 94$)		p
	M1	M2	M3	M4	M5	Med	(Q1, Q3)	Med	(Q1, Q3)		Med	(Q1, Q3)	
**Yes, but smear-negative**						21	(8.79)	20	(3.07)		0	(0)	
**Yes, and smear-positive**						11	(4.60)	2	(0.31)		35	(37.23)	
**Yes, and both smear-negative and smear-positive**						7	(2.93)	33	(5.06)		0	(0)	
**The prevalence of infectious patients in the cell by smear status (prevalence per 180 days)** †	-	√	√	-	√								
**Smear-negative**						0	(0, 0)	0	(0, 0)	-	0	(0, 0)	[a, b]
**Smear-positive**						0	(0, 0)	0	(0, 0)	-	0	(0, 0.02)	[a, b]
**The prevalence of infectious patients in the zone by smear status (prevalence per 100 persons per 180 days)** †	-	√	√	-	√								
**Smear-negative**						0.42	(0.32, 0.91)	0.82	(0.30, 0.82)	-	0	(0, 0)	[a, b]
**Smear-positive**						0.44	(0.21, 0.76)	0.51	(0.20, 0.51)	-	0.90	(0.57, 1.20)	[a, b]

M1, Wells–Riley model [7]; M2 and M3, Rudnick & Milton-proposed models (M2, ventilation rate in air changes per hour (ACH); M3, ventilation rate in liters/second/person (L/s/p)) [8, 9]; M4, the Applied Susceptible-Exposed-Infected-Recovered (SEIR) tuberculosis transmission model [10]; M5, the models proposed by Issarow *et al*. [11, 12]; Med (Q1, Q3), median (Quartile 1, Quartile 3)

[a] Statistical significance levels of less than 0.05 upon comparing Prisons A and B and Prisons A and C.

Wondershare PDFelement

[b] Statistical significance levels of less than 0.05 upon comparing Prisons B and C.

†A two-sample Wilcoxon rank–sum (Mann–Whitney) test was used to compare medians.

‡ A chi-squared or Fisher’s exact test.

Wondershare PDFelement

Wondershare PDFelement

TB transmission probability

Fig 1 illustrates the estimated TB transmission probability within cells using five prediction models. While the probability values varied obviously, the relative patterns of TB transmission probabilities among the three prisons were mostly comparable to those predicted by the Wells–Riley and the two Rudnick & Milton-proposed models. In particular, the highest transmission probabilities were observed in Prison A, while Prisons C and B had lower probabilities. The median TB transmission probabilities in the three prisons, as estimated by the Wells–Riley model, ranged from 0.036 to 0.100, while those estimated by the two Rudnick & Milton-proposed models ranged from 0.040 to 0.135 and 0.019 to 0.044. However, the models proposed by Issarow *et al*. and the Applied SEIR models predicted different TB transmission probabilities from other models, both in terms of values and relative patterns. Notably, the Applied SEIR model estimated TB transmission probabilities ranging from 0.157 to 0.570, with the highest probabilities observed in Prison C. Further analysis revealed that the correlations between the Applied SEIR model and the other models were the lowest. However, the correlations between the Wells–Riley model and the Rudnick & Milton (ACH) models were the highest (Fig 2).

Wondershare PDFelement

Fig 1. The estimated probability of tuberculosis transmission using five prediction models.

Fig 2. Spearman-rank correlations (ρ) between the five prediction models (*n* = 985, *p* <0.001 for all pairs).

Relationship between the model parameters and predicted probability of TB transmission

Wondershare PDFelement

The role of each parameter in differentiating the TB transmission probability was inferred from its progressive increase or decrease (i.e., dose–response pattern) based on the quartile of the TB transmission probability. The results showed that all parameters used in all models demonstrated a perfect or nearly perfect dose–response pattern (Table 2), particularly in the Rudnick & Milton (L/s/p) model, where four out of eight parameters demonstrated such property. The most distinct parameters showing a perfect dose–response pattern were “time-to-TB diagnosis” in four out of four models, “prevalence of TB cases in cell/zone: overall, smear-positive, and smear-negative TB cases together” in five out of five models, and 'number of inmates in the cell showing such property” in five out of five models.

Wondershare PDFelement

Wondershare PDFelement

Table 2. Quartiles of the cell-specific probability of tuberculosis transmission.

Local parameter	Quartiles of tuberculosis transmission probability										
	Q1		Q2		*p*‡	Q3		*p*‡	Q4		*p*‡
	Med	(Q1, Q3)	Med	(Q1, Q3)		Med	(Q1, Q3)		Med	(Q1, Q3)	
**The Wells–Riley model**											
(TB transmission probability)	(<0.0252)		(0.0252–0.0486)			(0.0487–0.1536)			(> 0.1536)		
Number of inmates in the cell	5.00	(5.00, 5.00)	6.00	(5.00, 12.00)	a	23.00	(8.00, 26.00)	ab	23.00	(11.00, 27.00)	ab
Number of overall TB cases in the cell §	0.50	(0.50, 0.50)	1.32	(0.50, 1.32)	a	0.98	(0.50, 1.32)	ab	2.15	(1.80, 2.15)	abc
Prevalence of overall TB cases in the cell	0	(0, 0)	0	(0, 0)	a	0	(0, 0)	ab	0	(0, 0.03)	abc
Prevalence of overall TB cases in the zone	0.50	(0.50, 0.50)	1.32	(0.50, 1.32)	a	0.98	(0.50, 1.32)	ab	2.13	(1.72, 2.15)	abc
Ventilation rate (ACH)	38.83	(26.99, 60.14)	48.31	(38.25, 56.45)	a	24.67	(18.49, 31.10)	ab	15.92	(12.93, 24.86)	abc
Time-to-TB diagnosis in the cell (months)	2.36	(2.36, 2.36)	3.88	(3.88, 5.50)	a	5.50	(3.88, 9.95)	ab	6.88	(3.88, 14.88)	abc
**The Rudnick & Milton-proposed models (ACH)**											
(TB transmission probability)	(<0.0240)		(0.0240–0.0480)			(0.0481–0.1590)			(> 0.1590)		
Number of inmates in the cell	5	(5, 9)	6	(5, 12)	a	22	(6, 32)	ab	23	(11, 26)	ab
Number of smear-negative patients in the cell §	0.30	(0.30, 0.30)	0.42	(0.32, 0.81)	a	0.33	(0.22, 0.81)	a	1.16	(0.91, 1.64)	abc
Number of smear-positive patients in the cell §	0.20	(0.20, 0.20)	0.51	(0.17, 0.51)	a	0.51	(0.44, 0.89)	ab	0.98	(0.80, 1.02)	abc

Wondershare PDFelement

Wondershare PDFelement

Local parameter	Quartiles of tuberculosis transmission probability										
	Q1		Q2		*p*‡	Q3		*p*‡	Q4		*p*‡
	Med	(Q1, Q3)	Med	(Q1, Q3)		Med	(Q1, Q3)		Med	(Q1, Q3)	
**Prevalence of smear-negative patients in the cell**	0	(0, 0)	0	(0, 0)	-	0	(0, 0)	ab	0	(0, 0)	abc
**Prevalence of smear-positive patients in the cell**	0	(0, 0)	0	(0, 0)	-	0	(0, 0)	ab	0	(0, 0)	ab
**Prevalence of smear-negative patients in the zone**	0.30	(0.30, 0.30)	0.42	(0.32, 0.82)	a	0.33	(0.22, 0.82)	a	1.16	(0.91, 1.64)	abc
**Prevalence of smear-positive patients in the zone**	0.20	(0.20, 0.20)	0.51	(0.17, 0.51)	a	0.51	(0.44, 0.89)	ab	0.98	(0.80, 0.98)	abc
**Ventilation rate (ACH)**	37.44	(27.69, 52.34)	48.58	(22.37, 59.08)	-	29.23	(9.21, 38.61)	ab	17.22	(3.72, 25.22)	abc
**Time-to-TB diagnosis in the cell (days)**	70.85	(70.85, 121.45)	116.39	(116.39, 164.97)	a	151.81	(16.39, 253.02)	ab	206.42	(16.39, 446.53)	abc
**Cell volume (m^3)**	26.98	(25.82, 55.08)	26.98	(26.98, 57.22)	a	100.80	(26.98, 115.81)	ab	100.80	(57.22, 113.67)	abc
**The Rudnick & Milton-proposed models (L/s/p)**											
**(TB transmission probability)**	(<0.0080)		(0.0080–0.0250)			(0.0251–0.0500)			(> 0.0500)		
**Number of inmates in the cell**	5	(5, 5)	11	(5, 23)	a	11	(6, 29)	ab	24	(11, 30)	abc
**Number of smear-negative patients in the cell §**	0.30	(0.30, 0.30)	0.33	(0.33, 0.81)	a	0.81	(0.22, 0.81)	ab	1.16	(0.91, 1.64)	abc
**Number of smear-positive patients in the cell §**	0.20	(0.20, 0.20)	0.21	(0.17, 0.51)	a	0.51	(0.51, 0.90)	ab	0.89	(0.76, 0.98)	abc

Wondershare PDFelement

Wondershare PDFelement

Local parameter	Quartiles of tuberculosis transmission probability										
	Q1		Q2		p‡	Q3		p‡	Q4		p‡
	Med	(Q1, Q3)	Med	(Q1, Q3)		Med	(Q1, Q3)		Med	(Q1, Q3)	
**Prevalence of smear-negative patients in the cell**	0	(0, 0)	0	(0, 0)	a	0	(0, 0)	a	0	(0, 0)	abc
**Prevalence of smear-positive patients in the cell**	0	(0, 0)	0	(0, 0)	a	0	(0, 0)	ab	0	(0, 0)	ab
**Prevalence of smear-negative patients in the zone**	0.30	(0.30, 0.30)	0.33	(0.33, 0.82)	a	0.82	(0.22, 0.82)	ab	1.16	(0.91, 1.64)	abc
**Prevalence of smear-positive patients in the zone**	0.20	(0.20, 0.20)	0.21	(0.17, 0.51)	a	0.51	(0.51, 0.90)	ab	0.89	(0.76, 0.98)	abc
**Ventilation rate (L/s/p)**	31.90	(26.53, 39.69)	26.80	(20.39, 35.14)	a	23.01	(19.55, 29.21)	ab	19.12	(15.25, 24.53)	abc
**Time-to-TB diagnosis in the cell (days)**	70.85	(70.85, 70.85)	164.97	(116.39, 164.97)	a	116.39	(116.39, 161.93)	ab	206.42	(116.39, 446.53)	abc
**Cell volume (m^3)**	26.98	(25.82, 26.98)	55.08	(25.82, 100.80)	a	57.22	(26.98, 115.81)	ab	100.80	(57.22, 115.81)	abc
**The Applied Susceptible-Exposed-Infected-Recovered (SEIR) tuberculosis transmission model**											
**(TB transmission probability)**	(<0.1783)		(0.1783–0.2202)			(0.2203–0.4105)			(> 0.4105)		
**Number of inmates in the cell**	5	(5, 5)	6	(5, 6)	a	22.5	(12, 25)	ab	25	(11, 49)	abc
**Number of overall TB cases in the cell §**	0.49	(0.49, 0.49)	1.32	(1.32, 1.32)	a	0.53	(0.50, 1.72	ab	2.12	(1.20, 2.15)	abc
**Prevalence of overall TB cases in the cell**	0	(0, 0)	0	(0, 0)	a	0	(0, 0)	ab	0	(0, 0.02)	abc
**Prevalence of overall TB cases in the zone**	0.49	(0.49, 0.49)	1.32	(1.32, 1.32)	a	0.53	(0.50, 1.72)	ab	2.08	(1.20, 2.15)	abc
**Ventilation rate (ACH)**	34.26	(25.87, 45.42)	48.30	(34.51, 57.51)	a	22.73	(16.86, 31.44)	ab	18.17	(13.83, 36.82)	abc

Wondershare PDFelement

Wondershare PDFelement

Local parameter	Quartiles of tuberculosis transmission probability										
	Q1		Q2		*p*‡	Q3		*p*‡	Q4		*p*‡
	Med	(Q1, Q3)	Med	(Q1, Q3)		Med	(Q1, Q3)		Med	(Q1, Q3)	
Area per person (m^3/person)	5.40	(5.16, 5.40)	5.16	(4.50, 5.40)	a	4.77	(4.38, 5.60)	a	4.58	(2.36, 5.51)	abc
Inmate turnover rate (%/year)	1.39	(1.39, 1.39)	1.69	(1.41, 1.69)	a	8.40	(4.64, 9.66)	ab	20.79	(8.38, 37.36)	abc
Transmission rate (persons/year)	0.025	(0.017, 0.035)	0.019	(0.015, 0.028)	a	0.041	(0.030, 0.055)	ab	0.054	(0.041, 0.079)	abc
**The models proposed by Issarow *et al*.**											
(TB transmission probability)	(<0.0030)		(0.0030–0.0110)			(0.0111–0.0750)			(> 0.0750)		
Number of inmates in the cell	5	(5, 5)	6	(5, 9)	a	12	(10, 24)	ab	28	(24, 42)	abc
Number of smear-negative patients in the cell §	0.30	(0.30, 0.30)	0.81	(0.33, 0.81)	a	0.33	(0.32, 1.16)	a	0.95	(0.00, 1.64)	abc
Number of smear-positive patients in the cell §	0.20	(0.20, 0.20)	0.51	(0.17, 0.51)	a	0.51	(0.21, 0.98)	ab	0.90	(0.81, 1.20)	abc
Prevalence of smear-negative patients in the cell	0	(0, 0)	0	(0, 0)	a	0	(0, 0)	a	0	(0, 0)	abc
Prevalence of smear-positive patients in the cell	0	(0, 0)	0	(0, 0)	a	0	(0, 0)	a	0	(0, 0.1)	abc
Prevalence of smear-negative patients in the zone	0.30	(0.30, 0.30)	0.82	(0.33, 0.82)	a	0.33	(0.32, 1.16)	a	0.91	(0.00, 1.64)	abc
Prevalence of smear-positive patients in the zone	0.20	(0.20, 0.20)	0.51	(0.17, 0.51)	a	0.51	(0.21, 0.98)	ab	0.89	(0.81, 1.20)	abc

Wondershare PDFelement

Wondershare PDFelement

**Local parameter**	**Quartiles of tuberculosis transmission probability**										
	**Q1**		**Q2**		**p‡**	**Q3**		**p‡**	**Q4**		**p‡**
	**Med**	**(Q1, Q3)**	**Med**	**(Q1, Q3)**		**Med**	**(Q1, Q3)**		**Med**	**(Q1, Q3)**	
**Ventilation rate (ACH)**	36.64	(26.81, 50.71)	43.40	(30.10, 55.66)	a	22.93	(15.29, 37.10)	ab	20.08	(13.86, 29.22)	abc
**Time-to-TB diagnosis in the cell (months)**	2.36	(2.36, 2.36)	3.88	(3.88, 5.50)	a	5.50	(3.88, 9.95)	ab	6.88	(5.06, 14.88)	ab

Med (Q1, Q3), median (Quartile1, Quartile 3)

‡ A two-sample Wilcoxon rank–sum (Mann–Whitney) test was used to compare medians.

[a] Statistical significance levels of less than 0.05 upon comparing Q1 and 2, Q1 and 3, and Q1 and 4.

[b] Statistical significance levels of less than 0.05 upon comparing Q2 and 3 and Q2 and 4.

[c] Statistical significance levels of less than 0.05 upon comparing Q3 and 4.

§ Sum of the prevalence of infectious patients in the cell (per 180 days) and in the zone (per 100 persons per 180 days).

Wondershare PDFelement

Wondershare
PDFelement

Furthermore, the impact of parameter changes on altering the TB transmission probability was investigated using multiple linear regression analysis. The results revealed that the magnitudes of altered probability were generally the highest across all the prediction models, particularly with categorical changes in ventilation of 4%–20% points and the number of TB cases in the zone of 1%–23% points, depending on the prediction model (Table 3). According to the Applied SEIR model, a marked alteration in TB transmission probability was observed for categorical changes in the 'number of inmates in cell" by 4%–12% points, the "area per person in square meter" by 11%–20% points, and the "inmate turnover rate" by 7%–22% points.

Wondershare
PDFelement

Wondershare PDFelement

Table 3. Multiple linear regression analysis of the association between model parameters and changes in tuberculosis transmission probability predicted by five prediction models (n = 985)

Cell characteristics	The Wells–Riley model		The Rudnick & Milton-proposed model (ACH)		The Rudnick & Milton-proposed model (L/s/p)		The Applied SEIR TB transmission model		Issarow *et al.*'s model	
	**Δtp**	**(95% CI)**	**Δtp**	**(95% CI)**	**Δtp**	**(95% CI)**	**Δtp**	**(95% CI)**	**Δtp**	**(95% CI)**
**Architectural characteristics**										
**Cell volume (m^2)**										
**<30.00**			Ref.		Ref.					
**30.01–60.00**			−0.02	(−0.02, −0.01)	−0.03	(−0.03, −0.02)				
**60.01–120.00**			−0.05	(−0.06, −0.04)	−0.05	(−0.05, −0.04)				
**> 120.00**			−0.08	(−0.09, −0.07)	−0.03	(−0.03, −0.02)				
**Ventilation rate (ACH)**										
**<12**	Ref.		Ref.				Ref.		Ref.	
**12.01–30.00**	−0.14	(−0.15, −0.12)	−0.05	(−0.06, −0.04)			−0.04	(−0.06, −0.02)	−0.11	(−0.12, −0.09)
**30.01–45.00**	−0.18	(−0.19, −0.16)	−0.08	(−0.09, −0.06)			−0.07	(−0.09, −0.04)	−0.13	(−0.14, −0.11)
**45.01–60.00**	−0.20	(−0.21, −0.18)	−0.09	(−0.10, −0.08)			−0.11	(−0.13, −0.08)	−0.14	(−0.15, −0.12)
**> 60.00**	−0.20	(−0.21, −0.18)	−0.10	(−0.11, −0.09)			−0.07	(−0.10, −0.04)	−0.15	(−0.16, −0.13)
**Absolute ventilation rate (L/s/p) †**										
**<15**					Ref.					
**16–30**					−0.05	(−0.06, −0.05)				

Wondershare PDFelement

Wondershare
PDFelement

Cell characteristics	The Wells–Riley model		The Rudnick & Milton-proposed model (ACH)		The Rudnick & Milton-proposed model (L/s/p)		The Applied SEIR TB transmission model		Issarow *et al.*'s model	
	**Δtp**	**(95% CI)**	**Δtp**	**(95% CI)**	**Δtp**	**(95% CI)**	**Δtp**	**(95% CI)**	**Δtp**	**(95% CI)**
**31–60**					−0.06	(−0.07, −0.06)				
**> 60**					−0.09	(−0.10, −0.08)				
**Demographic characteristics of cell inmates**										
**Number of inmates in the cell†**										
**≤5**	Ref.*		Ref.*		Ref.*		Ref.		Ref.	
**6–10**	−0.03	(−0.04, −0.02)	−0.03	(−0.03, −0.02)	−0.02	(−0.03, −0.02)	0.04	(0.02, 0.06)	−0.04	(−0.05, −0.03)
**11–15**	−0.01	(−0.03, 0.00)	−0.02	(−0.03, −0.01)	−0.02	(−0.03, −0.02)	0.11	(0.09, 0.13)	−0.03	(−0.04, −0.02)
**16–20**	−0.04	(−0.07, −0.01)	−0.04	(−0.06, −0.03)	−0.04	(−0.05, −0.02)	0.14	(0.09, 0.18)	−0.03	(−0.06, 0.00)
**21–25**	0.02	(−0.04, 0.00)	−0.05	(−0.06, −0.04)	−0.03	(−0.04, −0.02)	0.06	(0.04, 0.07)	0.01	(−0.01, 0.02)
**> 25**	0.00	(−0.01, 0.02)	−0.06	(−0.07, −0.05)	−0.03	(−0.04, −0.03)	0.12	(0.10, 0.14)	0.06	(0.05, 0.08)
**Area per person (m^3/person)**										
**≤3**	Ref.*		Ref.*		Ref.*		Ref.		Ref.*	
**3.01–4.00**	−0.01	(−0.04, 0.02)	0.01	(−0.01, 0.03)	0.01	(0.00, 0.03)	−0.11	(−0.15, −0.08)	−0.05	(−0.08, −0.03)
**4.01–5.00**	−0.03	(−0.04, −0.01)	0.04	(0.03, 0.05)	0.04	(0.03, 0.05)	−0.17	(−0.20, −0.15)	−0.06	(−0.08, −0.04)
**5.01–6.00**	−0.01	(−0.02, 0.01)	0.06	(0.04, 0.07)	0.04	(0.03, 0.06)	−0.20	(−0.22, −0.18)	−0.05	(−0.07, −0.03)
**> 6.00**	−0.03	(−0.04, −0.01)	0.05	(0.03, 0.06)	0.03	(0.02, 0.05)	−0.19	(−0.22, −0.17)	−0.07	(−0.09, −0.05)
**Inmate turnover rate (%/year)**										

Wondershare
PDFelement

Wondershare PDFelement

Cell characteristics	The Wells–Riley model		The Rudnick & Milton-proposed model (ACH)		The Rudnick & Milton-proposed model (L/s/p)		The Applied SEIR TB transmission model		Issarow *et al.*'s model	
	Δtp	(95% CI)	Δtp	(95% CI)	Δtp	(95% CI)	Δtp	(95% CI)	Δtp	(95% CI)
≤2							Ref.			
2.01–5.00							0.07	(0.05, 0.08)		
5.01–10.00							0.11	(0.09, 0.12)		
> 10							0.22	(0.20, 0.23)		
**Time-to-TB diagnosis in the cell (days)**										
≤100	Ref.		Ref.		Ref.		Ref.*		Ref.	
101–140	−0.04	(−0.05, −0.02)	−0.01	(−0.03, 0.00)	−0.01	(−0.02, 0.00)	0.00	(−0.02, 0.02)	−0.06	(−0.07, −0.05)
141–180	0.02	(0.00, 0.03)	0.01	(0.00, 0.02)	0.00	(−0.01, 0.01)	0.13	(0.11, 0.15)	0.00	(−0.01, 0.01)
> 180	0.08	(0.07, 0.10)	0.09	(0.08, 0.10)	0.05	(0.04, 0.06)	0.04	(0.03, 0.06)	0.02	(0.01, 0.03)
**Number of overall TB cases in the cell**										
0	Ref.		Ref.		Ref.		Ref.		Ref.	
1	0.03	(0.02, 0.05)	0.00	(−0.01, 0.01)	0.00	(−0.01, 0.01)	0.04	(0.02, 0.06)	0.03	(0.02, 0.05)
> 1	0.04	(0.02, 0.06)	0.02	(0.00, 0.03)	0.00	(−0.01, 0.01)	0.00	(−0.03, 0.02)	0.05	(0.04, 0.07)
**Number of TB cases in the cell by smear status†**										
No			Ref.		Ref.				Ref.	
Yes, but smear-negative			0.01	(−0.01, 0.02)	0.00	(−0.01, 0.01)			0.05	(0.03, 0.06)
Yes, and smear-positive			0.00	(−0.01, 0.02)	0.00	(−0.01, 0.01)			0.03	(0.01, 0.05)

Wondershare PDFelement

Cell characteristics	The Wells–Riley model		The Rudnick & Milton-proposed model (ACH)		The Rudnick & Milton-proposed model (L/s/p)		The Applied SEIR TB transmission model		Issarow *et al.*'s model	
	**Δtp**	**(95% CI)**	**Δtp**	**(95% CI)**	**Δtp**	**(95% CI)**	**Δtp**	**(95% CI)**	**Δtp**	**(95% CI)**
**Yes, and both smear-negative and smear-positive**			0.02	(0.01, 0.03)	0.01	(0.00, 0.02)			0.05	(0.03, 0.06)
**Prevalence of TB cases in the zone**										
**Smear-negative TB cases**										
**<0.30**			Ref.		Ref.				Ref.	
**0.30–0.50**			−0.10	(−0.11, −0.09)	−0.05	(−0.05, −0.04)			−0.07	(−0.09, −0.06)
**0.51–1.00**			0.02	(0.02, 0.03)	0.02	(0.02, 0.03)			0.01	(−0.01, 0.02)
**> 1.00**			0.05	(0.05, 0.06)	0.02	(0.01, 0.02)			0.03	(0.02, 0.04)
**Smear-positive TB cases**										
**<0.20**			Ref.		Ref.				Ref.	
**0.20–0.50**			0.02	(0.01, 0.04)	−0.02	(−0.03, −0.01)			0.01	(−0.01, 0.02)
**0.51–0.80**			0.06	(0.05, 0.07)	0.02	(0.02, 0.02)			0.03	(0.01, 0.04)
**> 0.80**			0.14	(0.13, 0.15)	0.07	(0.06, 0.07)			0.11	(0.09, 0.12)
**Smear-negative and smear-positive TB cases**										
**<0.50**	Ref.		Ref.		Ref.		Ref.		Ref.	
**0.50–1.00**	0.02	(0.00, 0.03)	0.03	(0.02, 0.04)	0.01	(0.00, 0.01)	0.11	(0.10, 0.13)	0.00	(−0.01, 0.01)
**1.01–1.70**	0.04	(0.03, 0.05)	0.06	(0.05, 0.07)	0.02	(0.02, 0.03)	0.02	(0.01, 0.03)	0.04	(0.03, 0.05)

Wondershare PDFelement

Wondershare PDFelement

Cell characteristics	The Wells–Riley model		The Rudnick & Milton-proposed model (ACH)		The Rudnick & Milton-proposed model (L/s/p)		The Applied SEIR TB transmission model		Issarow *et al*.'s model	
	**Δtp**	**(95% CI)**	**Δtp**	**(95% CI)**	**Δtp**	**(95% CI)**	**Δtp**	**(95% CI)**	**Δtp**	**(95% CI)**
**> 1.70**	0.20	(0.19, 0.21)	0.15	(0.14, 0.16)	0.07	(0.06, 0.07)	0.23	(0.22, 0.24)	0.09	(0.07, 0.10)

Δtp, magnitude of the change in the predicted TB transmission probability

* The parameter was not adjusted in the multiple linear regression model.

Wondershare PDFelement

Wondershare PDFelement

Discussion

In this study, five predictive models, i.e., the Wells–Riley model, two Rudnick and Milton-proposed models based on ACH and ventilation rate parameters (L/s/p), models proposed by Issarow *et al*., and the Applied SEIR model, were used to assess the probabilities of TB transmission in cells of three large Thai prisons. The results revealed that the median (Quartiles 1 and 3) of TB transmission probability among these cells was 0.052 (0.017, 0.180). Although the probability values differed, three models, i.e., the Wells–Riley model and the two Rudnick & Milton-proposed models, estimated similar patterns of TB transmission probability. The SEIR compartmental model estimated the highest TB transmission probability, particularly in Prison C compared to others. Further analysis revealed that the “ventilation rate” and the 'number of infectious TB patients in the cell/zone” had the greatest impact on the estimated TB transmission probability in most models. However, the 'number of inmates in the cell,” the “area per person in square meters,” and the “inmate turnover rate” were identified as high-impact parameters in the Applied SEIR model.

Wondershare PDFelement

The magnitude of TB transmission probability reported in our study differed from that reported in previous studies. Four previous studies utilized dynamic models to investigate TB transmission probability in prison settings. The first two studies, conducted by Johnstone-Robertson *et al*. in a South African prison and Cooper-Arnold *et al*. in a short-term urban lockup in Connecticut, USA, used the Wells–Riley model. Johnstone-Robertson *et al*. reported a TB transmission probability ranging from 5.4% to 90% [7], while Cooper-Arnold *et al*. reported a range of 0% to 15% [36]. In contrast, our study reported a TB transmission probability ranging from zero to 77%. These discrepancies may be attributed to the higher and

Wondershare PDFelement

wider range of ventilation rates and time-to-TB diagnosis reported in our study compared to Johnstone-Robertson *et al*.'s study (0–194 *vs.* 1–12 ACH and 71–507 *vs.* 0–180 days, respectively) [7]. However, Cooper-Arnold *et al*. reported ventilation rates of 1763 and 4954 cubic feet per minute for measuring and designing, respectively, or 22.48–63.19 L/s/p compared to 8.64–577.78 L/s/p in our study, and an exposure time of 19.1 hours per three days [36].

Urrego *et al*. conducted a study in Brazilian prisons using the Rudnick and Milton-proposed model and reported a TB transmission probability ranging from 14.2% to 99.9% in three prisons. Additionally, they reported the mean estimated TB transmission probabilities (95% confidence interval [CI]) of 78.9% (69.7–88.1), 81.1% (75.2–87.1), and 65.6% (54.6–76.5) for prisons in Estabelecimento Penal de Corumbá, Penitenciária de Três Lagoas, and Unidade Penal Ricardo Brandão, respectively [8]. In contrast, our study showed median (Quartiles 1 and 3) TB transmission probabilities of 13.5% (4.6, 25.7), 4% (2, 8.5), and 8.5% (3.2, 15.4) for Prisons A, B, and C, respectively, when the ventilation rate was assessed in ACH, and 0.04% (0.02, 0.10), 0.02% (0.01, 0.04), and 0.03% (0.02, 0.05), respectively, for absolute ventilation rate (L/s/p). These discrepancies may be attributed to the lower level of ventilation rate reported in their study, i.e., the mean (95% CI) of 9.5 (8.3–10.8), 23.9 (21.1–26.7), and 16.4 (11.9–20.8) L/s/p for the three studied prisons, compared to ours, i.e., the median (Quartiles 1 and 3) of absolute ventilation rates of 24.41 (18.57, 35.14), 24.53 (19.19, 30.86), and 33.99 (24.64, 48.60) L/s/p for Prisons A, B, and C, respectively.

The last study was conducted by Naning *et al*. on the probability of TB transmission in an overcrowded Malaysian prison using the SEIR model. They reported a TB transmission probability ranging from 0.83% to 8.80% [10], compared to 15.65% to 56.97% in our study.

Wondershare PDFelement

Wondershare PDFelement

This discrepancy may be attributed to our reported higher inmate turnover rate (0.28%–392.62% per year) versus Naning *et al.*'s study (33%–240% per year). However, no studies utilizing the models proposed by Issarow *et al*. have been reported in prison settings so far. Concerning the impactful parameters and interventions on TB transmission probability in prison settings, previous simulation studies revealed that these parameters included ventilation [7, 8, 10, 36], time-to-TB diagnosis [8], level of overcrowding [7, 10], active case-finding with increased ventilation and decreased lockup time [7, 37], and an appropriate treatment strategy with reduced overcrowding and increased ventilation [10, 27]. Among these, the "ventilation rate" and 'active case-finding together with other measures' were most frequently reported as high-impact parameters or interventions [37, 38]. In our study, all parameters used in the five prediction models were found to contribute significantly to the assessment of TB transmission probability. However, only the "ventilation rate" and 'number of infectious TB patients in the cell/zone" were shown to have a high impact on the predicted probability. Our finding of the "ventilation rate" as the highest-impact parameter in most predicting models aligns well with the existing evidence. In addition, our finding of the 'number of infectious TB patients in the cell/zone" as a high-impact parameter is implicitly consistent with the prevailing evidence on the impact of 'active case-finding" and 'appropriate treatment strategy." These measures, together with other measures, can reduce the number of active TB cases in the cell/zone. Therefore, these two parameters can be considered the most effective levers for designing interventions aimed at reducing TB transmission probability in prison settings. Importantly, our findings were based on actual data rather than simulations, including low-transmission-risk cells that serve as desirable local examples. This makes our findings practical and applicable in real-world settings, particularly within the context of Thai prisons.

Wondershare PDFelement

Wondershare PDFelement

Our finding of “number of inmates in the cell,” “area per person in square meters,” and 'inmate turnover rate” as high-impact parameters only in the Applied SEIR model is consistent with previous evidence about the “level of overcrowding” reported by Johnstone-Robertson *et al*. [7] and Naning *et al*. [10]. A high “inmate turnover rate” indicates that the number of TB-exposed inmates increased, leading to an increase in the number of TB-infected inmates. However, while a previous study [8] found “time-to-TB diagnosis” to be a high-impact parameter for TB transmission probability, such a finding was not observed in ours. This discrepancy may be due to our determining of the “time-to-TB diagnosis” parameter indirectly from health administrative data at the zone level of prisons rather than directly from each individual TB inmate, resulting in the inaccuracy of this parameter data.

It is worth noting that our study findings revealed a high variation in the magnitude and relative patterns of TB transmission probabilities estimated by the five dynamic models. Particularly, the Applied SEIR model showed low to moderate correlations with the other three models (ρ 0.33–0.57), but moderate to high correlations were observed among the other four models (ρ 0.60–0.90). This discrepancy may be attributed to the different theoretical concepts of the SEIR model compared to the other four models. The SEIR model is an epidemiological model that consists of compartments representing sets of individuals grouped by disease status. The link between compartments represents transitions from one disease state to another, and the TB transmission probability can be estimated by determining the model’s basic reproductive number [39]. In contrast, the Wells-Riley model and its three modified models similarly approach the airborne transmission of infectious disease as a physical transport problem, i.e., how an organism gets from one human body to another [40], with some minor differences. Both the Wells–Riley and Rudnick & Milton-proposed models quantify the

Wondershare PDFelement

Wondershare PDFelement

infectious dose based on the concept of 'quantum of infection,” although the former assumed steady-state ventilation conditions while the latter did not [7–9]. However, the models proposed by Issarow *et al*. are further modified by quantifying the infectious dose based on the infectors' average volume fraction of exhaled air using carbon dioxide level as a surrogate marker of exhaled air, as well as respiratory deposition fraction, and assuming either steady-state or nonsteady-state conditions [11–12]. Using the pioneered Wells–Riley model as a reference, the lower correlation between Issarow *et al*.'s and Wells–Riley's (ρ 0.78) models than that between Rudnick & Milton's (Q in ACH) and Wells–Riley's (ρ 0.91) models confirms the greater departure of the former model from the original Wells–Riley model than the latter model. This verifies the significant impact of the extensive modifications made in Issarow *et al.*'s model compared to Rudnick & Milton-proposed models (Q in ACH). However, when a different type of ventilation rate was used, as in the Rudnick & Milton-proposed model (L/s/p), the correlation between the Rudnick & Milton-proposed model (L/s/p) and the Wells–Riley model (ρ 0.72) markedly decreased. This may be due to the low agreement between the two types of ventilation rate measures (i.e., ACH *vs.* L/s/p), with a ρ value of only 0.33.

However, based on the previously mentioned evidence, the relative validity of these dynamic models could not be determined. Rather, they must be determined solely by their ability to predict TB incidence, which will be the focus of our upcoming report.

Strengths and limitations

This study has some advantages. First, it included five dynamic models, allowing for simultaneous examination and comparison of these models. Second, it had a large sample size (number of cells) and covered three large prisons with different architectural and administrative

Wondershare PDFelement

Wondershare PDFelement

characteristics and geographical locations. This ensures sufficient statistical power and generalizability of the study results. However, it has some limitations. First, the “time-to-TB diagnosis” parameter was derived indirectly from health administrative data at the prison zone level rather than directly from each individual TB inmate. Second, some TB progression parameters were obtained from the published literature rather than locally from the Thai population. These two limitations may have led to inaccuracies in estimating the TB transmission probability and evaluating the impact of these parameters on such a probability. Finally, the coronavirus disease 2019 pandemic that occurred during the study period may have affected some model parameters related to prison and health management. Therefore, the estimate of TB transmission probability may deviate from what would be expected during normal times. The pandemic, however, only occurred when the field data collection was almost complete.

Wondershare PDFelement

Conclusion

In this study, five different models were used to estimate TB transmission probabilities in 985 cells across three large Thai prisons. The probability patterns varied among the models. Compared to previous reports from countries with a similar developmental level, our estimated TB transmission probabilities were found to be generally lower than those reported in Brazil and South Africa, possibly due to the better ventilation in Thai prisons. However, our estimated TB transmission probabilities were higher than those reported in Malaysia, possibly due to our higher inmate turnover rate. In terms of risk factors, our study identified two parameters that significantly contribute to ongoing TB transmission risk in all models: low ventilation rates and a high number of existing TB inmates in the cell or zone. All stakeholders must urgently

In conclusion no need to mention comparative study with other countries

Wondershare PDFelement

address these issues to reduce TB transmission in prisons. Furthermore, since these five models produced varying estimates of TB transmission probabilities, further studies are required to determine their relative validity in accurately predicting TB incidence in prison settings.

Acknowledgments

The investigators would like to thank all stakeholders in the three prisons in this study for their assistance in data collection and survey. The investigators wish to thank the Department of Corrections at the Ministry of Justice, which gave permission for the study.

Wondershare PDFelement

Author Contributions

Conceptualization: Nithinan Mahawan and Wiroj Jiamjarasrangsi.

Data curation: Nithinan Mahawan and Wiroj Jiamjarasrangsi.

Formal analysis: Nithinan Mahawan, Wiroj Jiamjarasrangsi, Thanapoom Rattananupong, and Puchong Sri-Uam.

Funding acquisition: Wiroj Jiamjarasrangsi and Nithinan Mahawan.

Investigation: Nithinan Mahawan, Wiroj Jiamjarasrangsi, and Puchong Sri-Uam.

Methodology: Nithinan Mahawan, Wiroj Jiamjarasrangsi, Thanapoom Rattananupong, and Puchong Sri-Uam.

Project administration: Nithinan Mahawan and Wiroj Jiamjarasrangsi.

Resources: Nithinan Mahawan, Wiroj Jiamjarasrangsi, and Puchong Sri-Uam.

Software: Nithinan Mahawan, Wiroj Jiamjarasrangsi, and Thanapoom Rattananupong.

Wondershare PDFelement

Supervision: Nithinan Mahawan, Wiroj Jiamjarasrangsi, Thanapoom Rattananupong, and Puchong Sri-Uam.

Validation: Nithinan Mahawan, Wiroj Jiamjarasrangsi, Thanapoom Rattananupong, and Puchong Sri-Uam.

Visualization: Nithinan Mahawan and Wiroj Jiamjarasrangsi.

Writing–original draft: Nithinan Mahawan and Wiroj Jiamjarasrangsi.

Writing–review & editing: Nithinan Mahawan, Wiroj Jiamjarasrangsi, Thanapoom Rattananupong, and Puchong Sri-Uam.

References

1. World Health Organization. Use of high-burden country lists for TB by WHO in the post-2015 era Geneva: World Health Organization; 2015 [cited 2021 August 8]. Available from: https://www.who.int/tb/publications/global_report/high_tb_burdencountrylists2016-2020.pdf.
2. USAID. Tuberculosis in prisons: a growing public health challenge: USA; 2014 [cited 2021 August 8]. Available from: https://www.usaid.gov/sites/default/files/documents/1864/USAID-TB-Brochure.pdf.
3. World Health Organization. Tuberculosis. Key Facts Geneva2020 [cited 2021 August 8]. Available from: https://www.who.int/news-room/fact-sheets/detail/tuberculosis.

Wondershare PDFelement

Wondershare PDFelement

4. World Prison Brief. Highest to lowest - prison population total. [Internet]. UK. 2020 [cited 2022 February 25]. Available from: https://www.prisonstudies.org/highest-to-lowest/prison-population-total?field_region_taxonomy_tid=All.
5. Walter KS, Martinez L, Arakaki-Sanchez D, Sequera VG, Estigarribia Sanabria G, Cohen T, et al. The escalating tuberculosis crisis in central and South American prisons. Lancet. 2021;397(10284):1591-1596. doi: 10.1016/S0140-6736(20)32578-2
6. Mabud TS, de Lourdes Delgado Alves M, Ko AI, Basu S, Walter KS, Cohen T, et al. Evaluating strategies for control of tuberculosis in prisons and prevention of spillover into communities: an observational and modeling study from Brazil. PLOS Med. 2019;16(1):e1002737. doi: 10.1371/journal.pmed.1002737
7. Johnstone-Robertson S, Lawn SD, Welte A, Bekker LG, Wood R. Tuberculosis in a South African prison - a transmission modelling analysis. S Afr Med J. 2011;101(11):809-813.
8. Urrego J, Ko AI, da Silva Santos Carbone A, Paião DS, Sgarbi RV, Yeckel CW, et al. The impact of ventilation and early diagnosis on tuberculosis transmission in Brazilian prisons. Am J Trop Med Hyg. 2015;93:739-746.
9. Rudnick SN, Milton DK. Risk of indoor airborne infection transmission estimated from carbon dioxide concentration. Indoor Air. 2003;13(3):237-245. doi: 10.1034/j.1600-0668.2003.00189.x
10. Naning H, Al-Darraji HAA, McDonald S, Ismail NA, Kamarulzaman A. Modelling the impact of different tuberculosis control interventions on the prevalence of tuberculosis in an overcrowded prison. Asia Pac J Public Health. 2018;30(3):235-243. doi: 10.1177/1010539518757229

Wondershare PDFelement

Wondershare PDFelement

11. Issarow CM, Mulder N, Wood R. Modelling the risk of airborne infectious disease using exhaled air. J Theor Biol. 2015;372:100-106. doi: 10.1016/j.jtbi.2015.02.010

12. Issarow CM, Mulder N, Wood R. Environmental and social factors impacting on epidemic and endemic tuberculosis: a modelling analysis. R Soc Open Sci. 2018;5(1):170726. doi: 10.1098/rsos.170726

13. World Health Organization. WHO policy on TB infection control in health-care facilities, congregrate settings and households. Geneva2009.

14. Persily A. Evaluating building IAQ and ventilation with indoor carbon dioxide. ASHRAE Trans 103: 1–12. American Society of Heating, Refrigerating and Air-Conditioning Engineers (ASHRAE) annual meeting; 28 Jun - 2 Jul 1997; Boston, MA (United States). United States1997. p. 1-12.

15. World Health Organization. Natural ventilation for infection control in health-care settings. Atkinson J, Chartier Y, PessoaSilva CL, Jensen P, Li Y, Seto WH, eds. Geneva, Switzerland: World Health Organization; 2009 August 7, 2022. 133 p.

16. Pinna GD, Maestri R, La Rovere MT, Gobbi E, Fanfulla F. Effect of paced breathing on ventilatory and cardiovascular variability parameters during short-term investigations of autonomic function. Am J Physiol Heart Circ Physiol. 2006;290(1):H424-H433. doi: 10.1152/ajpheart.00438.2005

17. Guo Y, Qian H, Sun Z, Cao J, Liu F, Luo X, et al. Assessing and controlling infection risk with Wells-Riley model and spatial flow impact factor (SFIF). Sustain Cities Soc. 2021;67:102719. doi: 10.1016/j.scs.2021.102719

Wondershare PDFelement

Wondershare PDFelement

18. Issarow CM, Wood R, Mulder N. Seminal mycobacterium tuberculosis in vivo transmission studies: reanalysis using probabilistic modelling. Mycobact Dis. 2016;6(3):217. doi: 10.4172/2161-1068.1000217

19. Riley RL, Wells WF, Mills CC, Nyka W, McLean RL. Air hygiene in tuberculosis: quantitative studies of infectivity and control in a pilot ward. Am Rev Tuberc. 1957;75(3):420-431. doi: 10.1164/artpd.1957.75.3.420

20. Catanzaro A. Nosocomial tuberculosis. Am Rev Respir Dis. 1982;125(5):559-562. doi: 10.1164/arrd.1982.125.5.559

21. Noakes CJ, Sleigh PA. Mathematical models for assessing the role of airflow on the risk of airborne infection in hospital wards. J R Soc Interface. 2009;6 Suppl 6:S791-S800. doi: 10.1098/rsif.2009.0305.focus

22. Nardell EA, Keegan J, Cheney SA, Etkind SC. Airborne infection. Theoretical limits of protection achievable by building ventilation. Am Rev Respir Dis. 1991;144(2):302-306. doi: 10.1164/ajrccm/144.2.302

23. Riley RL, Mills CC, O'Grady F, Sultan LU, Wittstadt F, Shivpuri DN. Infectiousness of air from a tuberculosis ward. Ultraviolet irradiation of infected air: comparative infectiousness of different patients. Am Rev Respir Dis. 1962;85:511-525. doi: 10.1164/arrd.1962.85.4.511.

24. Behr MA, Warren SA, Salamon H, Hopewell PC, Ponce de Leon A, Daley CL, et al. Transmission of Mycobacterium tuberculosis from patients smear-negative for acid-fast bacilli. Lancet. 1999;353(9151):444-449. doi: 10.1016/s0140-6736(98)03406-0

Wondershare PDFelement

Wondershare PDFelement

25. Vynnycky E, Fine PE. The natural history of tuberculosis: the implications of age-dependent risks of disease and the role of reinfection. Epidemiol Infect. 1997;119(2):183-201. doi:10.1017/s0950268897007917

26. Dye C, Garnett GP, Sleeman K, Williams BG. Prospects for worldwide tuberculosis control under the WHO DOTS strategy. Directly observed short-course therapy. Lancet. 1998;352(9144):1886-1891. doi: 10.1016/s0140-6736(98)03199-7

27. Legrand J, Sanchez A, Le Pont F, Camacho L, Larouze B. Modeling the impact of tuberculosis control strategies in highly endemic overcrowded prisons. PLOS ONE. 2008;3(5):e2100. doi: 10.1371/journal.pone.0002100

28. Dowdy DW, Dye C, Cohen T. Data needs for evidence-based decisions: a tuberculosis modeler's 'wish list'. Int J Tuberc Lung Dis. 2013;17(7):866-877. doi: 10.5588/ijtld.12.0573

29. Blower SM, McLean AR, Porco TC, Small PM, Hopewell PC, Sanchez MA, et al. The intrinsic transmission dynamics of tuberculosis epidemics. Nat Med. 1995;1(8):815-21. doi: 10.1038/nm0895-815

30. Ministry of Health Malaysia, Academy of Medicine Malaysia. Clinical practice guidelines. Management of tuberculosis. 4, editor: Malaysian Health Technology Assessment Section (MaHTAS). Medical Development Division, Ministry of Health Malaysia; 2021. 97 p.

31. Grzybowski S, Enarson DA. The fate of cases of pulmonary tuberculosis under various treatment programmes. Bull IUAT. 1978;53:70-75.

Wondershare PDFelement

Wondershare
PDFelement

32. Noakes CJ, Beggs CB, Sleigh PA, Kerr KG. Modelling the transmission of airborne infections in enclosed spaces. Epidemiol Infect. 2006;134(5):1082-1091. doi: 10.1017/S0950268806005875

33. Carbone S, Paião DS, Sgarbi RV, Lemos EF, Cazanti RF, Ota MM, et al. Active and latent tuberculosis in Brazilian correctional facilities: a cross-sectional study. BMC Infect Dis. 2015;15:24. doi: 10.1186/s12879-015-0764-8

34. Storla DG, Yimer S, Bjune GA. A systematic review of delay in the diagnosis and treatment of tuberculosis. BMC Public Health. 2008;8:15. doi: 10.1186/1471-2458-8-15

35. Tiemersma EW, van der Werf MJ, Borgdorff MW, Williams BG, Nagelkerke NJ. Natural history of tuberculosis: duration and fatality of untreated pulmonary tuberculosis in HIV negative patients: a systematic review. PLOS ONE. 2011;6(4):e17601. doi: 10.1371/journal.pone.0017601

36. Cooper-Arnold K, Morse T, Hodgson M, Pettigrew C, Wallace R, Clive J, et al. Occupational tuberculosis among deputy sheriffs in Connecticut: a risk model of transmission. Appl Occup Environ Hyg. 1999;14(11):768-776. doi: 10.1080/104732299302198

37. Haeusler IL, Torres-Ortiz A, Grandjean L. A systematic review of tuberculosis detection and prevention studies in prisons. Glob Public Health. 2022;17(2):194-209. doi:10.1080/17441692.2020.1864753

38. Ndeffo-Mbah ML, Vigliotti VS, Skrip LA, Dolan K, Galvani AP. Dynamic models of infectious disease transmission in prisons and the general population. Epidemiol Rev. 2018;40(1):40-57. doi: 10.1093/epirev/mxx014

Wondershare
PDFelement

Wondershare PDFelement

39. Ozcaglar C, Shabbeer A, Vandenberg SL, Yener B, Bennett KP. Epidemiological models of Mycobacterium tuberculosis complex infections. Math Biosci. 2012;236(2):77-96. doi: 10.1016/j.mbs.2012.02.003

40. Sze To GN, Chao CY. Review and comparison between the Wells-Riley and dose-response approaches to risk assessment of infectious respiratory diseases. Indoor Air. 2010;20(1):2-16. doi:10.1111/j.1600-0668.2009.00621.x

Supporting Information Captions

S1 Table. Parameters used in prediction models

S2 Table. Models used in predicting the probability of tuberculosis transmission

Wondershare PDFelement

Fig 1. The estimated probability of tuberculosis transmission using five prediction models.

Title of figure should be down

Click here to access/download
Wondershare PDFelement

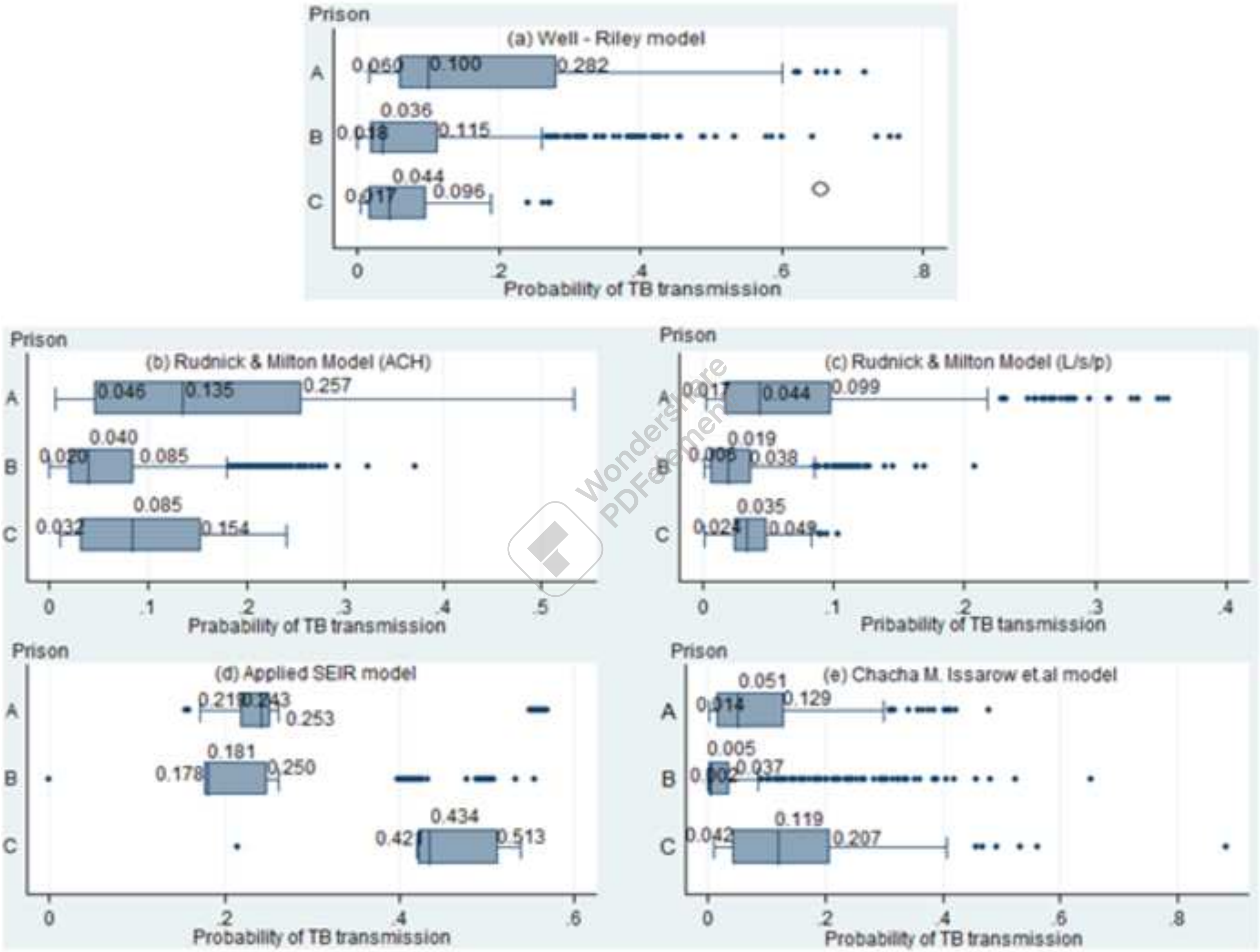

Wondershare PDFelement

Fig 2. Spearman-rank correlations (ρ) between the five prediction models (n = 985, p <0.001 for all pairs).

Click here to access/download
Wondershare PDFelement

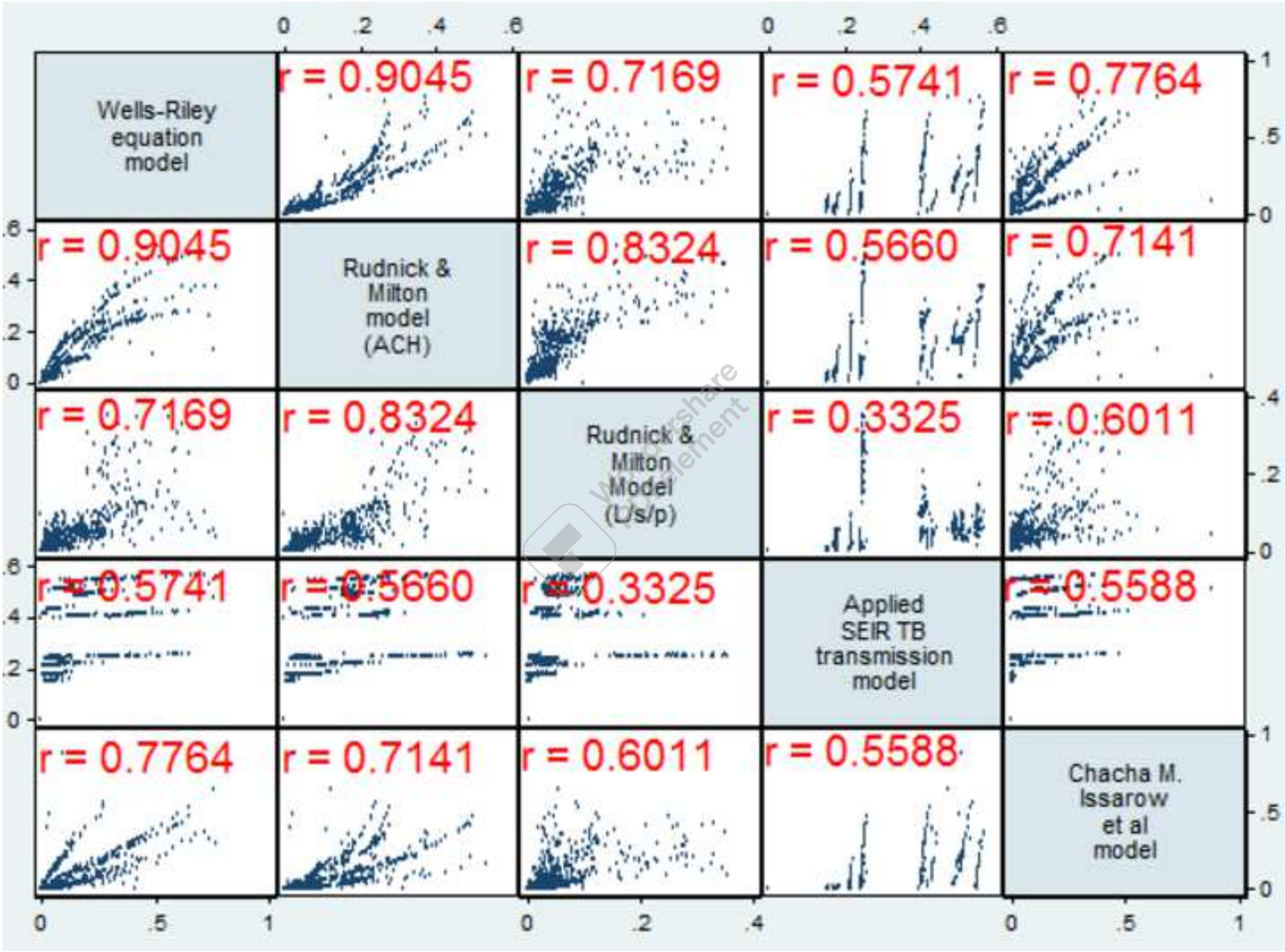

Wondershare
PDFelement

Click here to access/download
Supporting Information
July S2_Table-2.docx

Wondershare
PDFelement

Parameters used in prediction models

Wondershare PDFelement

Click here to access/download
Supporting Information
July_S1_Table-1.docx

Wondershare PDFelement
