# Supplementary material for: Assessment of tuberculosis transmission probability in three Thai prisons based on five dynamic models
Source: PLoS One. 2024 Jul 19;19(7):e0305264. doi: 10.1371/journal.pone.0305264 (PMC11259261; doi:10.1371/journal.pone.0305264)
Supplement: Attachment — Submitted filename: Response to Reviewers.docx [file pone.0305264.s006.docx]

**Response to academic editor** **and reviewers**

Thank you for the insightful comments provided by the editor and reviewers. We hope that our responses adequately address these comments and substantially improve the quality of our manuscript. Here are our responses:

**Academic Editor Zewdu Gashu Dememew, M.D, PhD**

| **Comment** | **Response** |
| --- | --- |
| 1.The abstract section was not well summarized. | Thanks for your comment. As per your suggestion, we revised our main manuscript as follows:   (1) We clarified our three study objectives, as shown on Page 2, Lines 26–27, and, Lines 30–31. (2) Moreover, we conducted additional statistical analyses to assess the agreement among the dynamic models, using the pioneering Wells–Reley’s equation as the reference. Details of this analysis are as follows: Analytical procedure on Page 11, Lines 228–233. Results on Pages 17–18, Lines 294–309. Discussion on Page 26, Lines 345–348. Conclusion on Pages 31–32, Line 468–471.   Furthermore, we revised the abstract according to the revisions made in the main manuscript, as shown on Page 2, Line 33-34, and Pages 2–3, Lines 37–41. |
| 2. The discussion section should have summarized the study key finding and the comparator studies should also be briefly summarized, rather than listing out their study findings in number/percentage/CI | We summarized the key findings of our study in the first paragraph of the “Discussion,” as shown on Page 26, Lines 345–352.  To enhance reader comprehension, we divided the “Discussion” into three subsections corresponding to the three study objectives, as follows: Pages 26–28, Lines 354–385; Pages 28–29, Lines 387–409; Pages 29–30, Lines 411–445.  Additionally, we prepared brief summaries of the comparator studies, as shown on Pages 26–28, Lines 357–385; Pages 28–29, Lines 397–409; and Pages 29–30, Lines 413–445. |
| 3. Revise the conclusion section for the study which should have summarized and interpreted the findings with future recommendation. The conclusion section looks like a discussion. | Thanks for your valuable comment. We have revised the conclusion accordingly (Pages 31–32, Lines 464–477). |

**Reviewer #1**

| **Comment** | **Response** |
| --- | --- |
| 1. Mention the type of sampling method. | Thank you for this insightful comment. We have added the type of sampling method in the study population section (Page 5, Lines 103–104). |
| 2. In conclusion no need to mention comparative study with other countries. | We have revised the conclusion, as per your suggestion (Pages 31–32, Lines 464–477). |

**Reviewer #2**

| **Comment** | **Response** |
| --- | --- |
| **Major comments** |  |
| Certain variables have been part of the data collection or at least obtained in similar settings. This includes for pulmonary ventilation rate, the relapse rate, partial acquired immunity after primary infection for treated persons, the rate of recovery under antituberculosis treatment, and TB-related death rate. Inferring those data from published study may not be a valid input data. | We have mentioned this limitation on Page 31, Lines 451–455. However, other factors that utilize actual values and demonstrate statistically significant relationships with the likelihood of tuberculosis transmission in each model of this study are worth considering for potential improvements to prevent and control tuberculosis in Thai prisons. Furthermore, it is important to verify the accuracy of the probabilities associated with each mentioned factor in future studies. (Reference: PONE-D-24-03144 “Validity of five dynamic models of predicting tuberculosis incidence in three prisons in Thailand” currently under Review). |
| Define "susceptible inmates" and state where the information about their health was obtained and how it was done. | Due to the COVID-19 situation, the actual follow-up period of this study lasted approximately 2 years, during which there were periods of high-inmate turnover rates in environments with high incidences and prevalence of tuberculosis. Moreover, research studies collecting data in actual prison contexts have been limited. Additionally, details regarding the health of the inmates were obtained from incomplete secondary data stored in each prison zone. Therefore, we did not utilize this data for analysis. Consequently, in this study, we define all inmates in the sample cell as susceptible inmates.  We have addressed this limitation on Page 31, Lines 457–461. |
| It is necessary to specify the TB case definition, including the diagnosis methods. | We have included this information as you suggested on Page 8, Lines 176–180. |
| The incidence of TB at Zone and cell was inferred using data obtained from documents and computer databases. Does the influence of under diagnosed and under reporting is considered? | The data were mainly secondary, comprising documents and computer databases sourced from the respective zonal and central administrative and medical facilities within the prisons. Although these data may be underreported, they reflect the actual situation within Thai prisons. However, we cross-checked the data obtained from central administrative and medical facilities with the data from each zone to ensure consistency for all PTB cases. |
| The time-to-TB diagnosis is determined using the time the individual diagnosed by x-ray to treatment initiated, but the required time is the time exposure prior to diagnosis. Please clarify. | We have mentioned this limitation on Page 31, Lines 451–453. |
| Include the details of how the number of recovered patients and mortality rates is determined. Does this information utilized as input data? A summary of the input data used for each model is more informative. | A summary of the input data used for each model is presented in **S2 Table**. **The models used for predicting the probability of tuberculosis transmission are listed in Table 1, which includes information on cell architectural, environmental, and tuberculosis risk characteristics in the three prisons studied (Page 13, Line 270).** We have also provided the details of how the number of recovered patients and mortality rates are determined in Table **S1, entitled “Parameters used in prediction models**.” |
| The variation in input parameters may account for the differences in the median TB transmission probability among the models. What is the rationale to utilize the different model with varied input parameters? | The rationale for using different models with varied input parameters was twofold: (a) to examine the agreement among TB transmission probabilities assessed using five dynamic models, and (b) to determine the impact of model parameters on TB transmission probability. These objectives were specified in the revised study objectives on Pages 5, Lines 95–99. |
| **Minor comments** |  |
| An introduction that includes an overview of the model’s description; justification for applying each the model i.e an explanation of its benefits and limitations. | Thank you for your comment. We have provided an overview of the models’ description on Page 4, Lines 75–88, which involved relocating some content from the “Discussion.”  Furthermore, we provide justification for the simultaneous application of all these models on Page 5, Lines 89-94. This approach allows us not only to determine their predictive validity simultaneously but also aligns with the objectives of our other study titled “PONE-D-24-03144-Validity of five dynamic models of predicting tuberculosis incidence in three prisons in Thailand” (currently under Review).  Notably, we have submitted two articles related to TB transmission in prison settings. Combining them into a single manuscript might make it lengthy and complicated. Therefore, separating them into two manuscripts seems appropriate. |
| The average occupancy rates have to be move from result section to the discussion. | According to your suggestion, we have moved such data from the “Materials and Methods” section to the “Discussion” (Page 30, Lines 433–435). |
| Discus about the key message for the TB program in the prison settings. | To facilitate better readability, we have divided the “Discussion” into three subsections aligned with the three study objectives on Pages 26–28, Lines 354–385; Pages 28–29, Lines 387–409; Pages 29–30, Lines 411–445  We have also summarized the key message of this study in our revised conclusion (Page 32, Lines 471–477). |
